# Bisphosphonate-induced differential modulation of immune cell function in gingiva and bone marrow *in vivo*: Role in osteoclast-mediated NK cell activation

**DOI:** 10.18632/oncotarget.4755

**Published:** 2015-07-28

**Authors:** Han-Ching Tseng, Keiichi Kanayama, Kawaljit Kaur, So-Hyun Park, Sil Park, Anna Kozlowska, Shuting Sun, Charles E. McKenna, Ichiro Nishimura, Anahid Jewett

**Affiliations:** ^1^ Division of Oral Biology and Medicine, The Jane and Jerry Weintraub Center for Reconstructive Biotechnology, UCLA School of Dentistry, Los Angeles, CA, USA; ^2^ Division of Advanced Prosthodontics, The Jane and Jerry Weintraub Center for Reconstructive Biotechnology, UCLA School of Dentistry, Los Angeles, USA; ^3^ Department of Periodontology, Asahi University School of Dentistry, Gifu, Japan; ^4^ Department of Tumor Immunology, Poznan University of Medical Sciences Poznan, Poland; ^5^ Department of Chemistry, University of Southern California, Los Angeles, CA, USA

**Keywords:** Immunology and Microbiology Section, Immune response, Immunity, osteoclasts, bisphosphonate, NK cell, zoledronate, etidronate

## Abstract

The aim of this study is to establish osteoclasts as key immune effectors capable of activating the function of Natural Killer (NK) cells, and expanding their numbers, and to determine *in vivo* and *in vitro* effect of bisphosphonates (BPs) during NK cell interaction with osteoclasts and on systemic and local immune function. The profiles of 27 cytokines, chemokines and growth factors released from osteoclasts were found to be different from dendritic cells and M1 macrophages but resembling to untreated monocytes and M2 macrophages. Nitrogen-containing BPs Zoledronate (ZOL) and Alendronate (ALN), but not non-nitrogen-containing BPs Etidronate (ETI), triggered increased release of pro-inflammatory mediators from osteoclasts while all three BPs decreased pit formation by osteoclasts. ZOL and ALN mediated significant release of IL-6, TNF-` and IL-1β, whereas they inhibited IL-10 secretion by osteoclasts. Treatment of osteoclasts with ZOL inhibited NK cell mediated cytotoxicity whereas it induced significant secretion of cytokines and chemokines. NK cells lysed osteoclasts much more than their precursor cells monocytes, and this correlated with the decreased expression of MHC class I expression on osteoclasts. Intravenous injection of ZOL in mice induced pro-inflammatory microenvironment in bone marrow and demonstrated significant immune activation. By contrast, tooth extraction wound of gingival tissues exhibited profound immune suppressive microenvironment associated with dysregulated wound healing due to the effect of ZOL which could potentially be responsible for the pathogenesis of Osteonecrosis of the Jaw (ONJ). Finally, based on the data obtained in this paper we demonstrate that osteoclasts can be used as targets for the expansion of NK cells with superior function for immunotherapy of cancer.

## INTRODUCTION

The bone resorptive and remodeling function of osteoclasts have been known for a long time, however, it is only recently that their function in the regulation of immune cell function has received attention [1]. Osteoclasts are known to initiate normal bone remodeling during bone growth, tooth eruption and fracture healing and also are able to mediate bone loss in pathologic conditions, such as osteoarthritis and osteoporosis. Osteoclasts are multinuclear giant cells derived from myeloid lineage [2, 3].

It has been well documented that Natural Killer (NK) cells participate in the clearance of virus-infected and transformed cells, as well as targeting cancer stem cells [4, 5]. The function and the role of NK cells in osteoblast regulation and bone remodeling are not well understood presently. IFN-γ, produced by both NK cells and Th1 lymphocytes, has been shown to inhibit osteoclastogenesis *in vitro* [6]. However, the *in vivo* effects of IFN-γ on bone tissue are less clear since many studies often provide a contrasting effect when compared to *in vitro* studies [7, 8]. TNF-α, another key cytokine produced by NK cells, can increase RANKL expression and RANKL dependent osteoclastogenesis [9–11]. NK cells have also been identified within inflamed synovial fluid and express RANKL and M-CSF, which during their interaction with monocytes can trigger the generation of osteoclasts [12].

Bisphosphonates (BPs) have become the treatment of choice for a variety of bone diseases in which excessive osteoclastic activity is one of the underlying pathological effects governing the disease, including Paget's disease of the bone, metastatic and osteolytic bone disease, hypercalcemia of malignancy and osteoporosis [13]. Etidronate (ETI) was the first BPs to be used in humans. Currently there are at least eleven BPs, which have been registered for various clinical applications in different countries. It was not until the 1990s that the biochemical actions of BPs were elucidated [14]. BPs are classified into two groups. Non-nitrogen-containing BPs, such as ETI and Clodronate are able to generate a toxic analog of adenosine triphosphate, which effectively inhibit the key function of mitochondria leading to the loss of energy production in osteoclasts. Nitrogen-containing BPs, such as Zolendronate (ZOL) and Alendronate (ALN), inhibit key enzymes of the mevalonate/cholesterol biosynthetic pathway. The major enzyme target for nitrogen-containing BP is farnesyl pyrophosphate synthase (FPPS). Inhibition of FPPS prevents the biosynthesis of isoprenoid compounds notably farnesol and geranylgeraniol that are required for the post-translational prenylation of small GTP-binding proteins such as rab, rho and rac, which are essential for intracellular signaling events within osteoclasts [14].

BPs are known to regulate the osteoclast-mediated bone resorptive activity in a variety of ways including osteoclast recruitment, differentiation and apoptosis [15–19]. Characteristic morphological feature of BP-treated osteoclasts is the lack of a ruffled border, the region of invaginated plasma membrane facing the resorptive cavity. BPs were also shown to disrupt the cytoskeleton of the osteoclast [20]. It is widely accepted that BPs exert their major effect on mature osteoclasts, however, *Kimachi et al.* suggested that nitrogen-containing BPs not only inhibit mature osteoclasts but also prevent osteoclast precursors from differentiating and migrating towards inflammatory osteolytic lesions [21]. It was also shown that BPs inhibit in a dose-dependent manner the formation of osteoclast-like cells in long-term cultures of human bone marrow cells [22].

Osteonecrosis of the Jaw (ONJ) is a severe bone disease that affects the maxilla and the mandible [23]. ONJ is commonly associated with BP therapy whereas other anti-resorptive agents are recently reported to also cause ONJ. The clinical manifestations of ONJ vary significantly from asymptomatic small fistulation to painful swelling with extensive bone exposure leading to pathological bone fracture [24–26].

As indicated above, the role of osteoclasts in bone remodeling is well established. However, their significance as member of the immune repertoire with a key role in regulation of both innate and adaptive immune cell function is not well understood and is the subject of this paper. Although the role of monocytes and dendritic cells (DCs) in the regulation of NK, T and γδ T cell function have received considerable attention previously [27–31], fewer reports have shown the significance of osteoclast interaction with these cells. Particularly, very little is known regarding the mode of BP-mediated modulation of NK, T and γδ T cell function by osteoclasts. In this paper we demonstrate that osteoclasts are potent activators of NK, T and γδ T cell function, and their effect is indeed more potent than monocytes and even DCs in the regulation of cytokine and chemokine secretion by NK cells. In addition, BP-treated osteoclasts activate the function of immune effectors, in particular NK cells in such a way, which may have the potential to establish chronicity of inflammation leading to pathologies observed in ONJ patients. We also demonstrate that depending on the site of action BPs have distinct effects on local immune cell function, being activating in the bone marrow but demonstrating significant suppression in the oral microenvironment where ONJ occurs.

## RESULTS

### Phenotypic and functional characterization of osteoclasts generated from purified human monocytes

Human osteoclasts were generated using purified monocytes treated with M-CSF and RANKL as described in the Materials and Methods section. The analysis of cytokines, chemokines and growth factors using multiplex cytokine array demonstrated a gradual increase in the secretion of IL-1RA, IL-2R, IL-12 cytokines and MIP-1α, MIP-1β and RANTES chemokines, whereas a decrease in IL-6 cytokine secretion can be observed from day 2 to day 21 of differentiation of monocytes to osteoclasts (Supplemental Table 1). Increased detection of IL-15 and IFN-α, but not IFN-γ, was also observed (Supplemental Table 1). The levels of MCP-1 and IL-8 remained significantly high at all time points tested. No significant secretion of IL-1β, IL-2, IL-4, IL-5, IL-7, IL-13, IL-17 and Eotaxin was observed at the time points and concentration tested (data not shown). In contrast to gradual decrease in IL-6 secretion from day 3 to day 16 of culture, IL-10 secretion in osteoclast precursors exhibited a gradual increase from day 3 to day 16 (Fig. S1). Thus, there was an inverse modulation of IL-6 and IL-10 during differentiation of osteoclasts from monocyte precursors.

### Differential induction of cytokines, chemokines and growth factors by monocytes, M1 and M2 macrophages, dendritic cells and osteoclasts

The profile and amounts of cytokines and chemokine secretion by osteoclasts resembled those of the freshly purified monocytes and cultured M2 macrophages, and were greatly distinct from M1 macrophages and dendritic cells (DCs) (Supplemental Table 2). M1 macrophages demonstrated the highest secretion of cytokines followed by the DCs, which had lower overall secretion for the majority of cytokines tested, although there were some exceptions such as IL-1Ra, which was higher by DCs (Supplemental Table 2). The amounts of cytokines were largely similar between freshly isolated monocytes, M2 macrophages and osteoclasts, with osteoclasts having the lowest secretion (Supplemental Table 2). Interestingly, the levels of chemokines were high in all the subsets, M1 macrophages having the highest for the MIP-1α and MIP-1β and the lowest for MCP-1 (Supplemental Table 2). IL-8 secretion was the highest from M1 macrophages and M2 macrophages and monocytes had the next highest secretion, whereas DCs and osteoclasts secreted lower amounts (Supplemental Table 2). Secretion of RANTES was the lowest for the M1 macrophages and higher in the other subsets (Supplemental Table 2). Monocytes, M2 macrophages and osteoclasts secreted the highest levels of MCP-1 and IP-10 when compared to DCs or M1 macrophages (Supplemental Table 2). Osteoclasts had the lowest amounts of growth factor secretion, whereas M1 macrophages had the highest with the exception of GM-CSF where they secreted the least (Supplemental Table 2). Overall, these results indicated that the profiles of cytokine, chemokine and growth factor secretion of osteoclasts resemble those of the monocytes and M2 macrophages and were distinct from DCs and M1 macrophages.

### Comparison of cell surface receptor expression between osteoclasts, monocytes, DCs and macrophages

When cell surface receptor expression was compared between osteoclasts and freshly isolated autologous monocytes, a significant down-modulation of all the cell surface receptors was observed on osteoclasts (Fig. S2A). Decreases in CD54, MHC-class I and II, CD44, CD14 and CD11b were more profound on the surface of osteoclasts when compared to freshly isolated autologous monocytes (Fig. S2A). However, when monocytes were cultured in media in the absence of RANKL and M-CSF and the levels were compared to osteoclasts, the differences between monocytes and osteoclasts decreased. The expression of all the cell surface receptors was significantly increased on the surface of macrophages as compared to either day 8 cultured monocytes or osteoclasts (Fig. S2B). DCs expressed higher levels of CD54, MHC-class II, CD44, CD11b, B7H1 and CD33 and lower levels of MHC class I and CD14 when compared to day 8 cultured monocytes or osteoclasts (Fig. S2B). When the surface expression of day 8 differentiated osteoclasts were compared with day 21, a slight increase in CD14, CD11b, B7H1 and CD33 and a decrease in MHC class II were noted (data no shown). The combinational addition of IFN-γ and TNF-α to mature osteoclasts decreased CD44 and increased CD54, MHC-class II, CD14, B7H1, CD33 and CD124, whereas no change in MHC-class I, CD11b and CD15 were noted (Fig. S2B).

### Osteoclasts were targets of NK cells and induced significant IFN-γ secretion by the NK cells

**Figure 1:**
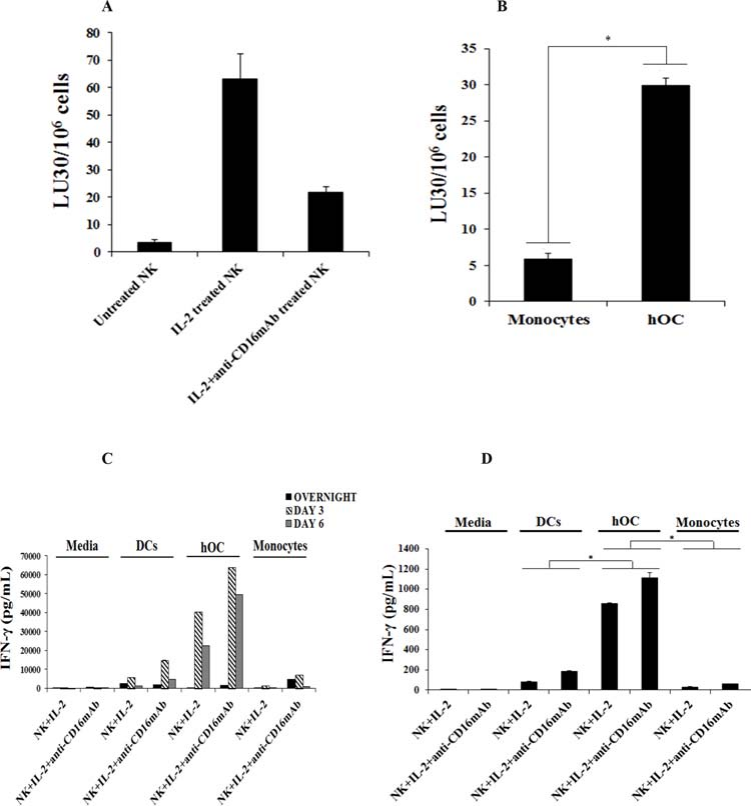

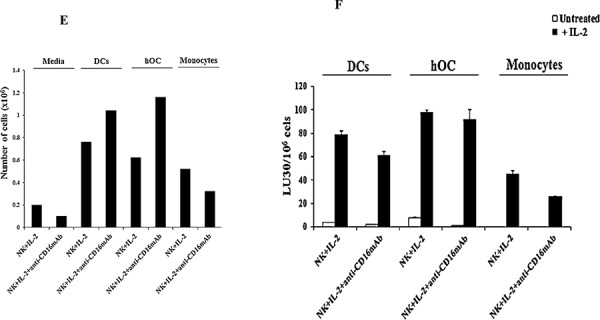
Osteoclasts are susceptible to NK cells mediated cytotoxicity and induce significant levels of IFN-γ secretion by the NK cells **A.** Highly purified NK cells (1 × 10^6^ cells/ml) were left untreated, treated with IL-2 (1000 units/ml) or a combination of IL-2 (1000 units/ml) and anti-CD16mAb (3 μg/ml) for 18 hours before they were added to ^51^Cr labeled autologous osteoclasts at various effector to target ratios. NK cell mediated cytotoxicities were determined using a standard 4 hour ^51^Cr release assay. **B.** NK cells were purified and treated with IL-2 (1000 units/ml) for 18 hours and then added to ^51^Cr labeled autologous monocytes or autologous osteoclasts (hOC) at various effector to target ratios. NK cell mediated cytotoxicities were determined using a standard 4 hour ^51^Cr release assay. * The difference between IL-2 treated NK cells cultured with monocytes or hOC is significant at *p* < 0.05. The lytic units 30/10_6_ cells were determined using inverse number of NK cells required to lyse 30% of the target cells X100. **C.** DCs, hOC and monocytes were prepared as described in Materials and Methods and seeded at 4 × 10^5^ cells/well in 12 well plates and incubated overnight. Purified NK cells were pre-treated with IL-2 (1000 units/mL) or a combination of IL-2 (1000 units/mL) and anti-CD16mAb hOCs for 18 hours and then cultured alone or with autologous DCs, hOC or monocytes at an effector to target ratio of 1:1. The supernatants from each culture condition were harvested overnight, day 3 and day 6. The level of IFN-γ produced by NK cells was measured using multiplex cytokine array kit. **D.** On the last day of the experimental period, the culture medium was refreshed and cells were cultured for an additional 5 hours. The level of IFN-γ produced by NK cells was measured with a specific ELISA. * The difference between IL-2 or IL-2+anti-CD16mAb treated NK cells cultured with DCs or monocytes compared to IL-2 or IL-2+anti-CD16mAb treated NK cells cultured with hOC is significant at *p* < 0.05 **E.** Monocytes, DCs and hOC were co-cultured with pre-treated NK cells, as described in Fig. 1C, and on the last day of the experimental period the number of cells was assessed by microscopic evaluation **F.** Pre-activated NK cells, as described in Fig. 1A, were co-cultured with autologous DCs, osteoclasts or monocytes for 6 days. Afterwards, the NK cells were divided into two groups-Untreated or treated with additional IL-2 (1000 units/ml) for 48 hours and used in a standard 4 hour ^51^Cr release assay against OSCSCs. The lytic units 30/10^6^ cells were determined using inverse number of NK cells required to lyse 30% of OSCSCs X100.

To determine whether osteoclasts are targets of NK cells, untreated, IL-2 treated or IL-2 in combination with anti-CD16mAb treated NK cells were used in cytotoxicity assays against osteoclasts. We have shown previously that the addition of anti-CD16mAb antibody in combination with IL-2 induced split anergy in NK cells resulting in the loss of cytotoxicity and an increase in IFN-γ secretion [33, 37, 38]. As shown in Fig. 1A, IL-2 treated NK cells were able to lyse osteoclasts significantly, and the addition of anti-CD16mAb in combination with IL-2 inhibited NK lysis as expected. To compare the levels of sensitivity to NK cell cytotoxicity between osteoclasts and monocytes, IL-2 treated NK cells were used in cytotoxicity assays. As shown in Fig. 1B, NK cells lysed osteoclasts significantly more than monocytes (*P* < 0.05).

In addition, both monocytes and osteoclasts were able to induce significant secretion of cytokines and chemokines by the NK cells, albeit the levels were much higher when NK cells were cultured with osteoclasts than monocytes (Table 1). Of note is significant up-regulation of IL-6, IL-10, MCP-1, MIP-1α and MIP-1β when osteoclasts were cultured with either IL-2 or IL-2+anti-CD16mAb treated NK cells as compared to cultures of NK cells either with monocytes or NK cells alone (Table 1). Indeed, 8–14 fold more cytokines and chemokines were secreted by the cultures of IL-2+anti-CD16mAb treated NK cells with osteoclasts when compared to those induced by cultures of monocytes with the NK cells (Table 1). We then compared the levels of IFN-γ secretion by IL-2 and IL-2+anti-CD16mAb treated NK cells cultured either with osteoclasts, DCs or monocytes from day 0 to day 6 of cultures (Fig. 1C). As shown in Fig. 1C, the levels of IFN-γ secreted in the cultures of NK cells with osteoclasts rose from day 0 to day 3 and remained high until day 6, and the secreted levels were much higher than those induced in the cultures of NK cells either with monocytes or DCs (*P* < 0.05) (Fig. 1D). Indeed, within 5 hours of culture NK cells secreted 5.5–27.7 folds more IFN-γ when cultured with osteoclasts than DCs (*P* < 0.05), and the levels were much less when cultured with monocytes (*P* < 0.05) (Fig. 1D). We then determined whether osteoclasts can support expansion of NK cells. As shown in Fig. 1E, both osteoclasts and DCs, and much less monocytes, were able to support the expansion of NK cells. NK cells expanded by osteoclasts, DCs and monocytes were counted and equal numbers of NK cells from each expanded subset were used in cytotoxicity assay against OSCSCs, which is a very sensitive NK cell target. IL-2 treated NK cells expanded by osteoclasts had the highest cytotoxicity, followed by those expanded by DCs and the least cytotoxicity was seen by IL-2 treated NK cells which were expanded by monocytes (Fig. 1F). NK cells in the absence of osteoclasts, DCs or monocytes did not expand, had much lower viability, and were not able to mediate cytotoxicity (data not shown).

**Table 1:** Production of cytokines, chemokines and growth factors in cultures of NK cells with monocytes and osteoclasts treated with or without ZOL

		IL-6	IFN-γ	IL-10	MCP-1	IL-8	MIP-1α	MIP-1β
**Media**	**Media alone**	7	1	0	56	7	1039	0
	**Untreated NK alone**	24	1	0	999	1514	942	375
	**IL-2 treated NK alone**	54	4	0	1252	3638	1039	586
	**IL-2 + anti-CD16mAb treated NK alone**	252	25	0	3818	16969	5803	20368
**Monocytes**	**Untreated**	106	4	0	239	11217	1377	375
	**+ Untreated NK**	99	2	0	264	10426	1135	163
	**+ IL-2 treated NK**	136	4	10	1232	11125	1232	374
	**+ IL-2 + anti-CD16mAbtreated NK**	424	33	10	28195	21638	1619	4229
**Osteoclasts**	**Untreated**	41	4	14	31691	16607	1377	2129
	**+ Untreated NK**	63	4	23	29258	16544	1715	2185
	**+ IL-2 treated NK**	535	4	24	38655	19349	1908	5680
	**+ IL-2 + anti-CD16mAbtreated NK**	3866	57	82	38916	23766	6114	61555
**Osteoclasts + ZOL**	**Untreated**	196	4	14	37820	18589	1425	2157
	**+ Untreated NK**	312	6	14	38159	20986	1715	2538
	**+ IL-2 treated NK**	3267	12	20	39332	22631	4294	11587
	**+ IL-2 + anti-CD16mAb treated NK**	11605	80	31	38748	24420	6105	38411

Purified NK cells were left untreated or pre-treated with IL-2 (1000 units/mL) or the combination of IL-2 (1000 units/ml) and anti-CD16mAb (3 μg/ml) for 18–24 hours. Human monocytes purified from autologous donor's PBMCs were differentiated into osteoclasts as described in the Materials and Methods section for 17 days. Osteoclasts were then left untreated or treated with ZOL (1 μM) for 72 hours then washed extensively with fresh medium and lastly co-cultured with pre-treated NK cells for 24 hours. Afterwards, supernatants from each condition were harvested and the levels of cytokine and chemokine production were measured using multiplex cytokine array kit.

### Uptake of ZOL and ALN by osteoclasts

**Figure 2:**
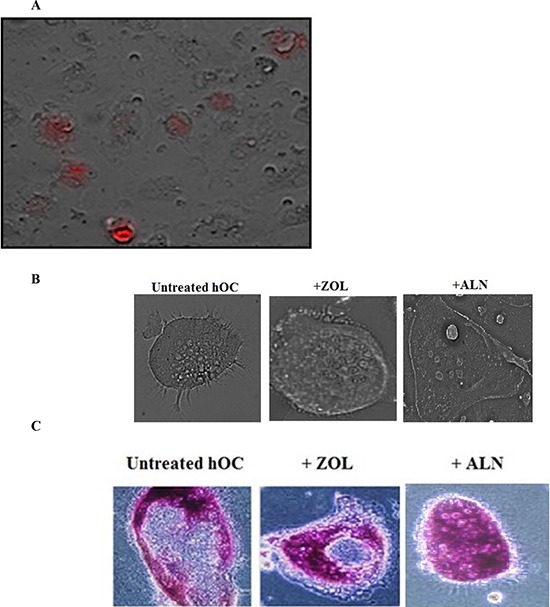
Uptake and the pro-inflammatory effect of ZOL and ALN on osteoclasts **A.** Human osteoclasts were generated from healthy donor's monocytes as described in the Materials and Methods section. Afterwards, osteoclasts were washed with 1X PBS, detached from the tissue culture plate and seeded at a density of 3 × 10^4^ cells/well in 24 well plate. After an overnight incubation, the cells were treated with ZOL conjugated with *5 - Carboxy - X - rhodamine* (5 μM) for 24 hours and the image was taken with Leica DMI 6000B microscope **B.** Osteoclasts were prepared as described in the Materials and Methods section and seeded at 1 × 10^4^ cells/well in 24 well plates. The osteoclasts were then treated with ZOL or ALN (1 μM) for 7 days. Afterwards, culture medium was removed and the cells were washed with 1X PBS and fixed with solution containing citrate solution, acetone and 37% formaldehyde for 5 minutes. The cells were then washed with water and photographed using Leica DMI 6000B inverted microscope **C.** Osteoclasts were prepared as described in the Materials and Methods section and seeded at 1 × 10^4^ cells/well in 24 well plates. The osteoclasts were then treated with ZOL or ALN (1 μM) for 7 days. Afterwards, culture medium was removed and the cells were rinsed with 1X PBS and fixed with solution containing citrate solution, acetone and 37% formaldehyde for 5 minutes. Cells were washed with twice with 1XPBS before Fast Garnet GBC and sodium nitrite at (1:1 ratio) were added and incubated for 1 hour at 37° (C) Plates were rinsed and hematoxylin were then added to each well for 2 mins. Plates were air dried and images were taken by the Leica DMI 6000B inverted microscope.

To determine the effect of BPs on osteoclasts, first we assessed the specific uptake of red-fluorescent-labeled ZOL (ROX-ZOL) by osteoclasts (Fig. 2A). As shown in the Fig. 2A, osteoclasts, which had taken up ROX-ZOL, appeared red under the microscope. Human osteoclasts differentiated from monocytes exhibit different sizes and shapes and demonstrate multinucleated morphology in the presence and the absence of BPs (Fig. 2B) and are TRAP positive (Fig. 2C).

### Decreased pit numbers and size by BP-treated osteoclasts

**Figure 3:**
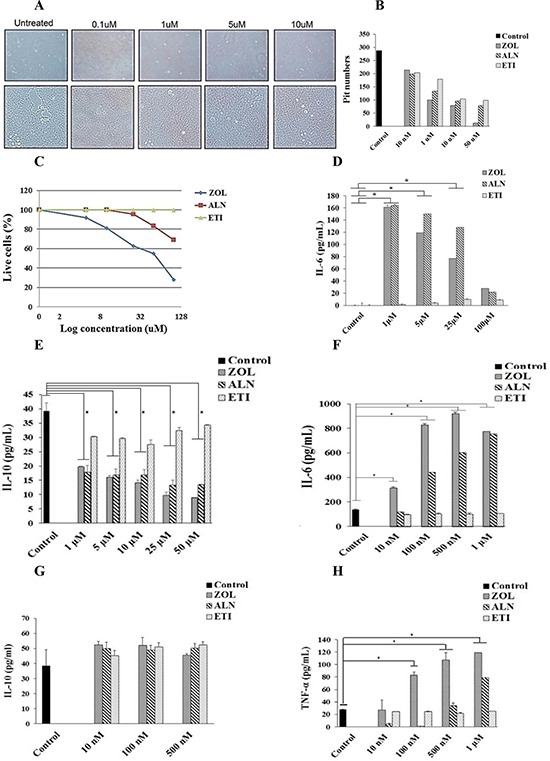
Decreased size, pit numbers, viability and cytokine secretion mediated by ZOL, ALN, and ETI-treated osteoclasts **A.** Osteoclasts were generated as described in the Materials and Methods section for 12 days. Osteoclasts (2.5 × 10^5^ cells/ml) were treated with different concentrations (10 nM-50 μM) of ZOL for 48 hours, and afterwards the cells were washed using freshly prepared 5% sodium hypochlorite and the images were taken with Leica DMI 6000B inverted microscope at 5X magnification **B.** Osteoclasts were prepared as described in Fig. 3A and treated with varying concentrations (10 nM-50 μM) of ZOL, ALN or ETI for 48 hours. Afterwards, the number of pits was counted using microscopy at 5X magnification **C.** Purified osteoclasts were treated with varying concentrations of ZOL, ALN and ETI for 48 hours, afterwards the cells were washed with 1X PBS, stained with propidium iodide and analyzed by flow cytometry **D.** Osteoclasts were generated as described in the Materials and Methods section. After the differentiation period, osteoclasts were washed with 1X PBS, detached from tissue culture plates and seeded at 1.5 × 10^4^ cells/well in 24 well plate for 24 hours. Cells were then left untreated or treated with ZOL, ALN or ETI at 1, 5, 10, 25, 50 or 100 μM for 6 days. Supernatants were removed on the last day of experiment and the levels of IL-6 **E.** and IL-10 **F.** were determined with specific ELISAs. * The difference between osteoclasts treated with ZOL or ALN compared to untreated osteoclasts is significant at *P* < 0.05. Osteoclasts were generated as described in the Materials and Methods section for 12 days. Afterwards, osteoclasts were seeded at 2 × 10^5^ cells/ml in 24 well plate for 24 hours, and treated with ZOL, ALN or ETI at 10 nM, 100 nM, 500 nM or 1 μM for 3 days after which the supernatants were harvested and the levels of IL-6 **G.** IL-10 **H.** and TNF-α (I). were determined with specific ELISAs. * The difference between osteoclasts treated with ZOL or ALN compared to untreated osteoclasts is significant at *P* < 0.05.

The resorptive activity of the osteoclasts was determined after treatment with ZOL at 0.1–10 uM concentration. A dose dependent decrease in the resorptive activity of osteoclasts could be seen by the treatment with ZOL (Fig. 3A). As shown in Fig. 3B, the effect of resorptive activity by osteoclasts was severely hindered when treated with ZOL, as compared to ALN and ETI (Fig. 3B). The numbers of the pits were affected by the treatment with the three BPs (Fig. 3B). We then determined the effect of all three BPs on osteoclasts' cell viability. As shown in Fig. 3C there was a dose dependent increase in cell death when nitrogen-containing BPs, ZOL and ALN, were added to osteoclasts, with ZOL having higher toxicity than ALN (Fig. 3C). The non-nitrogen containing BP, ETI, did not mediate cell death at any concentration (Fig. 3C).

### ZOL and ALN, but not ETI, modulated cytokine secretion of osteoclasts

ZOL and ALN, but not ETI, were able to increase the secretion of IL-6 at the level of 1 μM (*P* < 0.05) and the levels significantly decreased with the 25–100 μM concentration of ZOL (*P* < 0.05). ALN at the levels of 100 μM exhibited significant decrease in IL-6 secretion when compared to 1–25 μM levels (*P* < 0.05) (Fig. 3D). ETI at all concentrations was not able to modulate the secretion of IL-6 (Fig. 3D). In contrast to elevated secretion of IL-6 by nitrogen-containing BPs, the levels of anti-inflammatory cytokine IL-10 were severely suppressed at the concentrations of 1–50 μM (*P* < 0.05) (Fig. 3E). ETI at all concentrations had the ability to increase or retain the secretion of IL-10 by osteoclasts (Fig. 3E).

Since the highest increase in IL-6 secretion was observed at 1 μM, we then determined the ability of lower concentrations of BPs to influence the IL-6 secretion at the range of 10 nM to 1 μM (Fig. 3F). The results demonstrated a dose and time dependent increase in IL-6 secretion by ZOL and ALN (*P* < 0.05), with ZOL having higher ability to induce IL-6 (Fig. 3D and 3F). ETI had no or minimal effect on the secretion of IL-6 at all concentrations (Fig. 3D and 3F). ZOL and ALN at concentrations below or at 500 nM did not have a significant effect on IL-10 secretion; however, they inhibited IL-10 secretion at the concentrations of 1–50 μM (*P* < 0.05) (Fig. 3E and 3G). Dose dependent increase in TNF-α (Fig. 3H) and IL-1β (data not shown) secretion was also observed by ZOL (*P* < 0.05), followed by ALN (*P* < 0.05) and no secretion by ETI. Overall, these data demonstrated the ability of both ZOL and ALN, but not ETI, to induce pro-inflammatory cytokines, whereas they had inhibitory effect on the release of anti-inflammatory cytokine IL-10 (Fig. 3).

### ZOL-modulated surface receptor expression on osteoclasts

**Figure 4:**
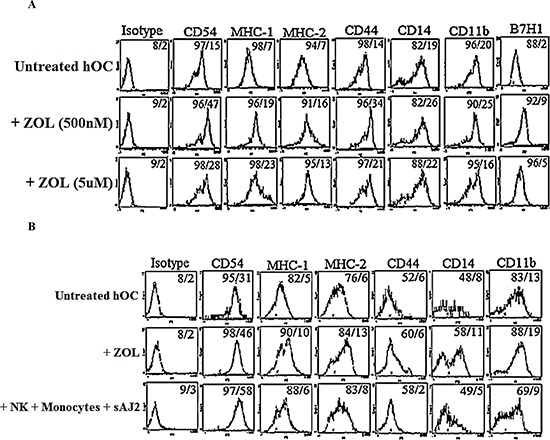
Osteoclasts treated with supernatants from activated NKs or ZOL demonstrated modulation of cell surface receptors **A.** Osteoclasts were generated as described in the Materials and Methods section for 17 days. Osteoclasts were then left untreated or treated with ZOL at 500 nM or 5 μM. After 4 days of treatment, surface expression of CD54, MHC-I, MHC-II, CD44, CD14, CD11b and B7H1 were determined using flow cytometric analysis after staining with the respective PE-conjugated antibodies **B.** Osteoclasts were left untreated or treated with ZOL (1 μM) or supernatants harvested from pre-activated NK cells cultured with monocytes and probiotic bacteria (sAJ2) for 48 hours. Surface expressions of CD54, MHC-I, MHC-II, CD44, CD14 and CD11b were determined using flow cytometric analysis after staining with the respective PE-conjugated antibodies **C.** Isotype control antibodies were used as control. The numbers on the right hand corner are the percentages and the mean channel fluoresence intensities for each histogram.

Treatment of osteoclasts with ZOL increased all cell surface receptors at lower concentration of ZOL, which correlated with the increased cytokine induction (Figs. 3 and 4A). At higher concentration of ZOL there was less increase in the cell surface receptors (Fig. 4A). Comparison between ZOL-treated osteoclasts and those treated with the supernatants prepared from the activated NK cells demonstrated higher induction of cell surface receptors by ZOL with the exception of CD54 for which supernatant treated osteoclasts had higher induction (Fig. 4B). Of note both CD14 and CD44 expression were significantly down-modulated by NK supernatant treated osteoclasts (Fig. 4B).

### ZOL-treated osteoclasts were resistant to NK cell mediated cytotoxicity

**Figure 5:**
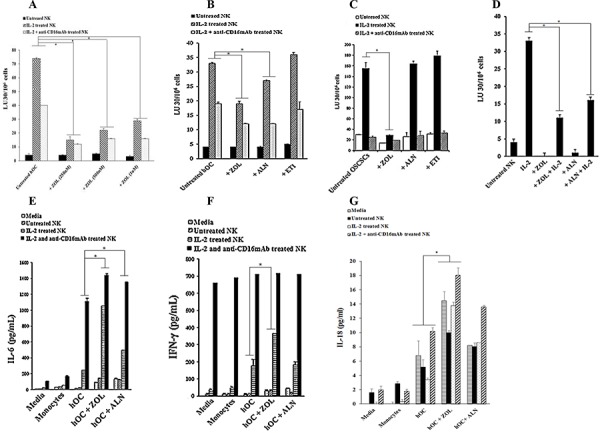
ZOL-treated osteoclasts and OSCSCs were resistant to NK-mediated cytotoxicity and secreted high levels of IFN-γ **A.** Highly purified NK cells (1 × 10^6^ cells/ml) were left untreated or treated with IL-2 (1000 units/ml) or a combination of IL-2 (1000 units/ml) and anti-CD16mAb (3 μg/ml) for 18 hours before they were added to ^51^Cr labeled osteoclasts (hOC) at various effector to target ratios. Osteoclasts were prepared as described in the Materials and Methods section and treated with ZOL (250 nM, 500 nM or 1 uM) for 30 minutes before they were used as target cells. NK cell mediated cytotoxicity was determined using a standard 4 hour ^51^Cr release assay. * The difference between IL-2 or IL-2+anti-CD16mAb stimulated NK cells treated with ZOL compared to IL-2 or IL-2+anti-CD16mAb stimulated NK cells without ZOL treatment is significant at *P* < 0.05 **B.** Osteoclasts were prepared as described in the Materials and Methods section and treated with ZOL, ALN or ETI (100 nM) for 30 minutes before used as target cells. NK cells were prepared as described in Fig. 5A and then added to ^51^Cr labeled osteoclasts at various effector to target ratios. NK cell mediated cytotoxicity was determined using a standard 4 hour ^51^Cr release assay. * The difference between IL-2 or IL-2+anti-CD16mAb stimulated NK cells treated with ZOL or ALN compared to IL-2 or IL-2+anti-CD16mAb stimulated NK cells without BP treatment is significant at *P* < 0.05 **C.** OSCSCs were treated with ZOL, ALN or ETI (1 μM) for 30 minutes before the addition of pre-treated NK cells, prepared as described in Fig. 5A. NK cell mediated cytotoxicity was determined using a standard 4 hour ^51^Cr release assay. * The difference between IL-2 stimulated NK cells treated with ZOL compared to IL-2 treated NK cells without BP treatment is significant at *P* < 0.05 **D.** Osteoclasts were differentiated from autologous monocytes as described in the Materials and Methods section for 17 days. NK cells were left untreated or treated with IL-2 (1000 units/ml) in the presence and absence of ZOL (500 nM) and ALN (500 nM) for 18 hours before they were added to ^51^Cr labeled osteoclasts at various effector to target ratios. NK cell mediated cytotoxicity was determined using a standard 4 hour ^51^Cr release assay. * The difference between IL-2 stimulated NK cells treated with ZOL or ALN compared to IL-2 stimulated NK cells without BP treatment is significant at *P* < 0.05. The lytic units 30/10^6^ cells were determined using inverse number of NK cells required to lyse 30% of the osteoclasts or OSCSCs X100. **E.** Osteoclasts were treated as described in Fig. 5B and then added to untreated, IL-2 treated or IL-2+anti-CD16mAb treated NK cells at 1:3 ratio (NK: target cells). After an overnight incubation, the supernatants were harvested and the levels of IL-6 **F.** IFN-γ **G.** and IL-18 were measured with specific ELISAs. * The difference between IL-2 or IL-2+anti-CD16mAb stimulated NK cells treated with ZOL or ALN compared to IL-2 or IL-2+anti-CD16mAb stimulated NK cells without BP treatment is significant at *P* < 0.05 (E) * The difference between IL-2 stimulated NK cells treated with ZOL compared to IL-2 stimulated NK cells without BP treatment is significant at *P* < 0.05 (F) * The difference between untreated, IL-2 or IL-2+anti-CD16mAb stimulated NK cells treated with ZOL compared to those NK cells without BP treatment is significant at *P* < 0.05.

We then determined the cytotoxic activity of NK cells against BPs-treated osteoclasts and BP-treated OSCSCs. IL-2 treated NK cells lysed untreated osteoclasts, while treatment with ZOL induced resistance in osteoclasts against NK cell cytotoxicity (*P* < 0.05) (Fig. 5A). The resistance to cytotoxicity is also seen with ALN (*P* < 0.05) (Fig. 5B). Osteoclasts treated with ETI were not resistant to NK cell cytotoxicity (Fig. 5B). To determine whether the ability to induce resistance in NK cells is specific for osteoclasts, we treated OSCSCs with ZOL for 15–30 min and used in cytotoxicity assay against NK cells. Treatment of OSCSCs with ZOL induced resistance against NK cytotoxicity, as seen in Fig. 5C (*P* < 0.05). Treatment of osteoclasts with ZOL and ALN for a longer period of time induced substantial resistance in osteoclasts against NK cell mediated cytotoxicity (*P* < 0.05) (Fig. 5D).

### NK cells secreted significant levels of inflammatory cytokines and chemokines in culture with ZOL-treated osteoclasts

We next determined the effect of BPs when NK cells were cultured with either ZOL- or ALN-treated osteoclasts. As shown in Table 1 and Fig. 5E–5G, ZOL-treated osteoclasts triggered significantly higher induction of cytokines and chemokines in the co-cultures with NK cells, and the effect was higher when compared to ALN-treated osteoclasts (*P* < 0.05) (Fig. 5E–5G), whereas ETI had no enhancing effect (data not shown). ZOL-treated osteoclasts upregulated secretion of cytokines and chemokines 2–6 fold higher for IL-6, IFN-γ, IL-18, MIP-1α and MIP-1β by IL-2 and IL-2+anti-CD16mAb treated NK cells when compared to untreated osteoclasts (*P* < 0.05) (Table 1 and Fig. 5E–5F). The levels of IL-8 and MCP-1 secretion were very high and plateaued in the cultures of NK cells with osteoclasts (Table 1). ZOL-treated osteoclasts demonstrated decreased secretion of IL-10 in the cultures with IL-2+anti-CD16mAb treated NK cells when compared to untreated osteoclasts (Table 1). ZOL-treated osteoclasts secreted higher levels of IL-18 when compared to monocytes (*P* < 0.05), and the levels changed moderately when cultured with NK cells (Fig. 5G).

### Similar to human osteoclasts ZOL-treated murine osteoclasts triggered significant secretion of IFN-γ by NK, CD3^+^ T cells and γδT cells

**Figure 6:**
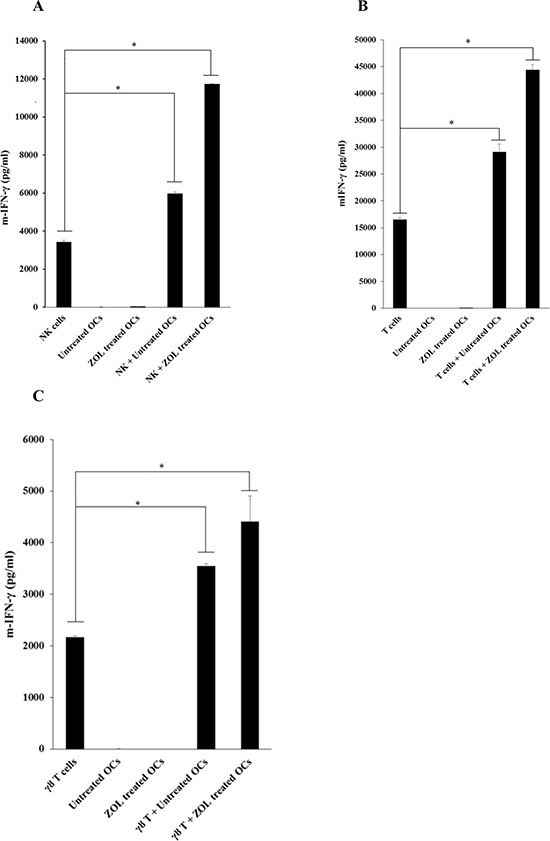
ZOL-treated murine osteoclasts trigger significant secretion of IFN-γ by NK, CD3^+^ T cells and γδ T cells **A.** Monocytes were purified from bone marrow cells extracted from femurs of WT B6 mice. Osteoclasts were generated as described in the Materials and Methods section, treated with or without ZOL (500 nM) for 12 hours and then washed extensively before they were cultured with pre-activated NK cells (1 × 10^6^ cells/ml) treated with IL-2 (10, 000 units/ml for 12 hours) at 2:1 ratio (NK cells: osteoclasts). At the time of the co-culture of NK cells with osteoclasts the cultures were supplemented with LPS (100 ng/ml) and incubated for 48 hours before they were washed and treated with only IL-2 (10, 000 units/ml) for an additional 48 hours, after which the supernatants were harvested and the levels of secreted IFN-γ were determined using specific ELISAs. * The difference between IL-2 treated NK cells cultured with untreated OCs or ZOL treated OCs compared to IL-2 treated NK cells cultured in media is significant at *P* < 0.05 **B.** Osteoclasts, and highly purified T cells were prepared and co-cultured as described for NK cells and co-cultured as described in Fig. 6A. * The difference between IL-2 treated T cells cultured with untreated OCs or ZOL treated OCs compared to IL-2 treated T cells cultured in media is significant at *P* < 0.05 **C.** Osteoclasts, and highly purified γδT cells were prepared and co-cultured as described for NK cells and co-cultured as described in Fig. 6A. * The difference between IL-2 treated γδT cells cultured with untreated OCs or ZOL treated OCs compared to IL-2 treated γδT cells cultured in media is significant at *P* < 0.05 (C) After 4 days of culture, the supernatants were harvested and the levels of IFN-γ were measured with specific ELISAs.

Similar to human osteoclasts, murine osteoclasts were also able to trigger the secretion of IFN-γ by murine NK cells (Fig. 6A). Significant induction of IFN-γ secretion by mouse osteoclasts in a single stimulation occurred after overnight incubation (data not shown) and continued until day 11, albeit the levels decreased from day 8 to day 11 (data not shown). Osteoclasts were also able to induce secretion of IFN-γ by CD3^+^ T cells (*P* < 0.05) and γδT cells (*P* < 0.05), however, differences in IFN-γ secretion between T cells with and without osteoclasts could only be seen after 4 days of incubation (Fig. 6B and Fig. 6C). Treatment of osteoclasts with ZOL was able to mediate significant increases in IFN-γ secretion by murine NK, CD3^+^ T cells and γδ T cells when compared to those cultured without osteoclasts or cultured with untreated osteoclasts (Fig. 6).

### Intravenous injection of ZOL in mice stimulated IFN-γ and IL-6 secretion by bone marrow derived cells but inhibited secretion by gingival cells

**Figure 7:**
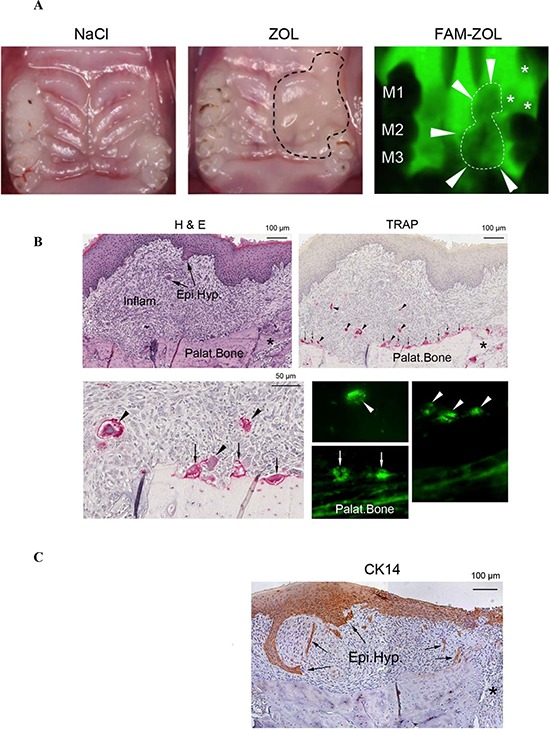

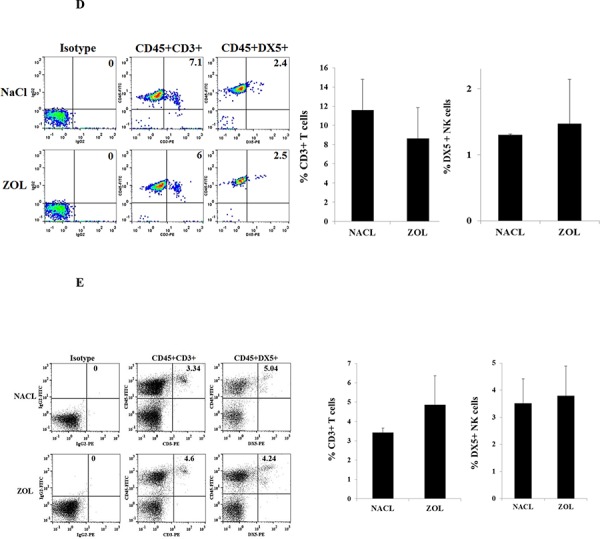

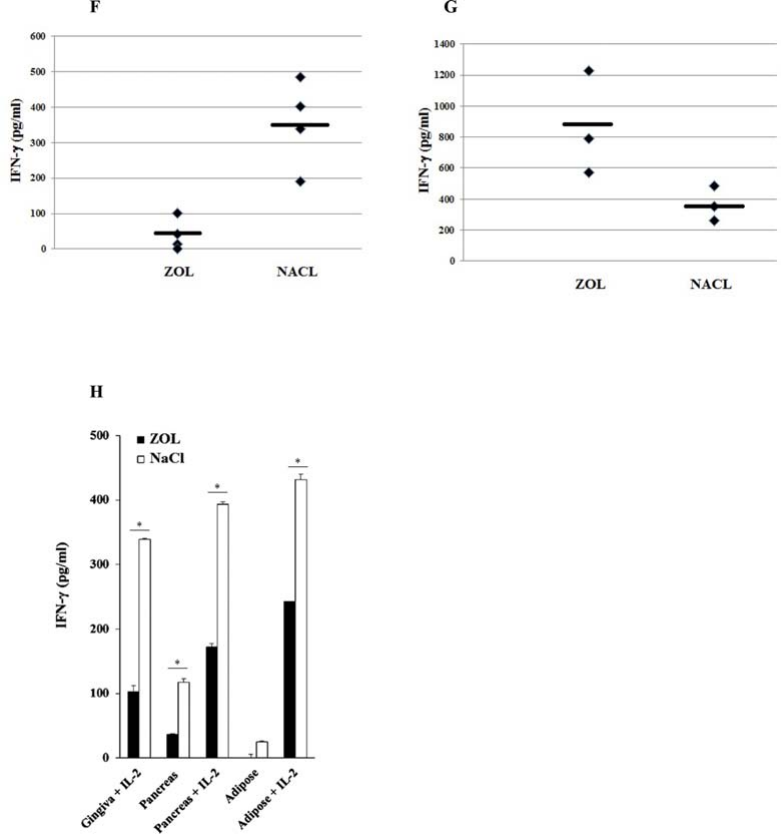

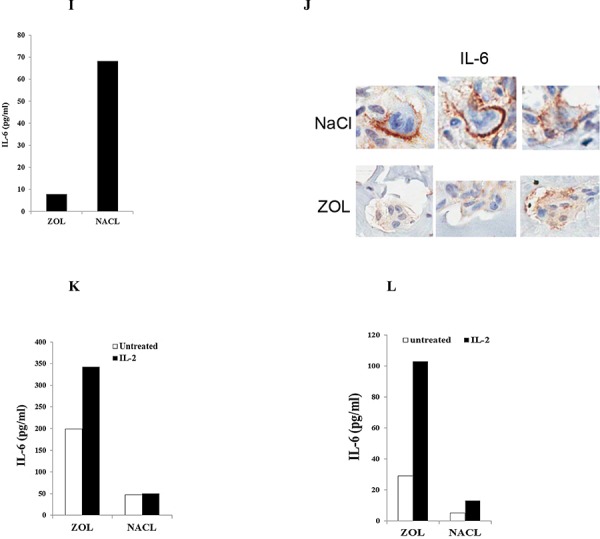

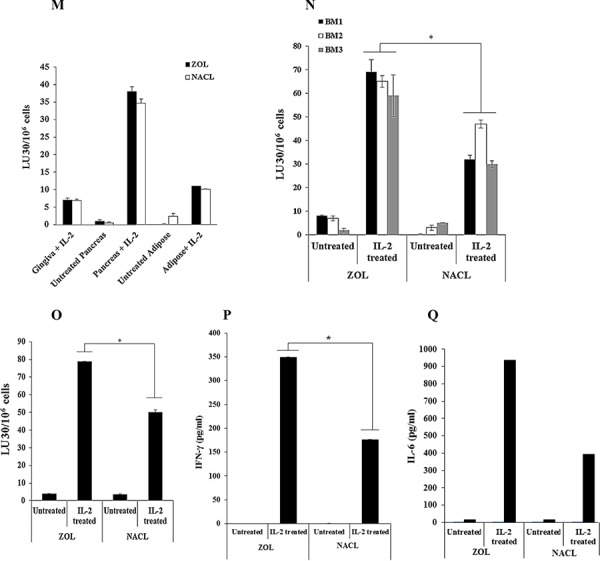

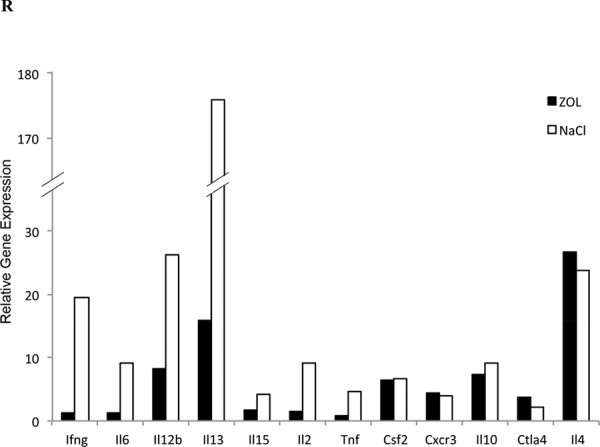
*In vivo* injection of ZOL triggered significant IFN-γ and IL-6 secretion by bone marrow-derived cells but inhibited secretion by gingival cells **A.** Female Balb/c mice received IV injection of 500 μg/Kg ZOL or 0.9% NACL vehicle solution followed by maxillary first molar extraction after 6 days. Two weeks after tooth extraction, the maxillary palatal mucosa of 0.9% NACL solution-injected control mice showed complete wound healing, whereas tissue swelling (dotted line) around the tooth extraction site remained in ZOL-injected mice. The maxillary bone of FAM-ZOL-injected mice showed the strong fluorescence signal, while the remaining molars (M1, M2, M3) were absent of fluorescence. The visibly reduced fluorescence was observed in the tooth extraction sockets (*) and the adjacent palatal bone area (dotted line and arrowheads) **B.** Hematoxylin and eosin (H & E) stained histological sections of ZOL-injected mice showed significant inflammatory cell infiltration (Inflam) in the palatal/gingival soft tissue one week after tooth extraction. There were signs of abnormal epithelial hyperplasia (Epi.Hyp.: arrows). The extraction socket (*) exhibited new bone formation. TRAP staining revealed a number of osteoclasts (vertical arrows) on the surface of palatal bone (Palat.Bone) as well as in the extraction socket (*). TRAP^+^ multinuclear cells were found within the inflammation area (arrowheads). In non-decalcified cross-section of FAM-ZOL-injected mice, FAM-positive large cells (arrows) were found on the palatal bone as well as away from the bone surface (arrowheads). Taken together, these cells in the inflammatory area were thought to be ZOL-affected osteoclasts **C.** Immunohistology for cytokeratin 14 (CK14) was performed as described in the Materials and Method section, and confirmed oral epithelial hyperplasia in the palatal/gingival tissue of ZOL-injected mice (Epi.Hyp.: arrows). Epithelial hyperplasia was primarily observed within the strong inflammatory area **D.** Female B6 mice received IV injection of 500 μg/Kg ZOL or 0.9% NACL vehicle solution followed by maxillary first molar extraction after 6 days. Two weeks after tooth extraction the surface expression of CD45^−^FITC^+^CD3^−^PE or CD45^−^FITC^+^DX5^−^PE on cells obtained from gingival tissues. **E.** and bone marrow **F.** were assessed with flow cytometric analysis after staining with the respective PE- and FITC-conjugated antibodies. Isotype control antibodies were used as control. Right upper quadrant represent the percentage of CD3 and DX5 positive cells within the CD45 population. B6 mice underwent experimental treatment as described in Fig. 7D. Gingival tissues obtained from ZOL and NACL injected animals were digested as described in Materials and Method. Dissociated cells obtained from gingival tissues were cultured in the presence of IL-2 (10,000 units/ml). Supernatants were harvested 5 days after IL-2 treatment. The mean for (*n* = 4) is shown for each set in the figure. The difference between ZOL and NACL injected gingivae is significant at a *P* < 0.05 **G.** After euthanasia, femurs from ZOL and NACL injected animals were harvested and bone marrow cells were extracted and cultured (1 × 10^6^ /ml) in the presence of IL-2 (10, 000 units/ml). Supernatants were harvested 5 days after IL-2 treatment. The mean for (*n* = **4**) is shown for each set in the figure. The difference between ZOL and NACL injected bone marrow cells is significant at a *P* < 0.05 **H.** Gingival tissue, pancreas and adipose (1 × 10^6^ /ml) from ZOL and NACL injected animals were cultured in the absence or presence of IL-2 (10, 000 units/ml) for 5 days, after which the supernatants were harvested and the levels of IFN-γ were measured with specific ELISA. *The difference between untreated or IL-2 stimulated cells from ZOL injected mice compared to untreated or IL-2 stimulated cells from NACL injected mice is significant at *P* < 0.05 **I.** Female WT B6 mice received IV injection of ZOL or NACL as described in Fig. 7A. After euthanasia, gingival tissues obtained from ZOL and NACL injected mice were digested as described in Materials and Methods section and cultured (1 × 10^6^ /ml) in the presence of IL-2 (10, 000 units/ml). After five days of culture, supernatants from the cultures of gingival tissue were harvested and the levels of IL-6 secretion were measured by specific ELISA. **J.** Immunohistology for IL-6 was performed as described in the Materials and Methods section, and showed the IL-6 immuno-localization pattern was distinctly was different in osteoclasts in the jawbone of 0.9% NACL solution-injected control mice and ZOL-injected mice **K.** Female B6 mice received IV injection of ZOL or NACL as described in Fig. 7A. After euthanasia, femurs from ZOL and NACL injected mice were harvested and bone marrow cells were extracted and cultured (1 × 10^6^ /ml) in the presence and absence of IL-2 (10,000 units/ml). After five days of culture, the supernatants were removed and the levels of IL-6 secretion were measured by specific ELISA. **L.** The pancreas was obtained from ZOL and NACL injected mice and digested as described in Materials and Methods section. Dissociated pancreatic cells were cultured in the presence and absence of IL-2 (10, 000 units/ml). After five days of culture, the supernatants were removed and the levels of IL-6 secretion were measured by specific ELISA **M.** Dissociated gingival, pancreas and adipose cells (1 × 10^6^ /ml) from ZOL and NACL injected animals were cultured in the absence or presence of IL-2 (10, 000 units/ml) for 5 days and used as effectors against ^51^Cr labeled ST63 cells at various effector to target ratios in a standard 4 hour ^51^Cr release assay. The lytic units 30/10^6^ cells were determined using inverse number of NK cells required to lyse 30% of the ST63 cells X100 **N.** Total cells from the bone marrow were prepared as described in Fig. 7K **O.** were prepared as described in Fig. 7K. NK cells were purified from bone marrow cells and cultured in the presence or absence of IL-2 (10, 000 units/ml) for 5 days **P.** Afterwards, the cells were used as effectors against ^51^Cr labeled ST63 cells at various effector to target ratios in a standard 4 hour ^51^Cr release assay. The lytic units 30/10^6^ cells were determined using inverse number of NK cells required to lyse 30% of ST63 cells X100. * The difference between IL-2 treated BM (N) or BM-NK (O) cells obtained from mice injected with ZOL compared to IL-2 treated BM or BM-NK cells from mice injected with NACL is significant at *P* < 0.05. NK cells purified from bone marrow were cultured in the presence and absence of IL-2 (10, 000 units/ml) after which supernatants were harvested at day 5 and the levels of IFN-γ **Q.** and IL-6 **R.** were determined by specific ELISAs. One week after tooth extraction, total RNA samples were isolated from palatal/gingival tissue of control and ZOL-injected mice. PCR microarray analysis revealed the suppressed expression of pro-inflammatory cytokines such as IFN-γ (ifng), IL-6, -12b, -15, -2 and TNF-α (Tnf) in the ZOL-injected mice. Those unaffected cytokines included anti-inflammatory cytokines such as IL-10 and -4 (R) (A complete list of PCR microarray data can be found in Fig. S3).

Mice were injected with ZOL and the effects were compared to 0.9% NACL solution injected mice. Two weeks after tooth extraction, the maxillary palatal mucosa of NACL-injected control mice showed complete wound healing, whereas tissue swelling (dotted line, Fig. 7A) around the tooth extraction site remained in ZOL-injected mice (Fig. 7A). The maxillary bone of FAM-ZOL-injected mice showed the strong fluorescence signal, while the remaining molars (M1, M2, M3) was absent of fluorescence (Fig. 7A). The visibly reduced fluorescence was observed in the tooth extraction sockets and the adjacent palatal bone area (dotted line and arrowheads) (Fig. 7A). As shown in Fig. 7B, 7H & 7E stained histological sections of ZOL-injected mice showed significant inflammatory cell infiltration in the palatal/gingival soft tissue one week after tooth extraction. There were signs of abnormal epithelial hyperplasia. The extraction socket showed new bone formation (Fig. 7B). TRAP staining revealed a number of osteoclasts on the surface of palatal bone as well as in the extraction socket (Fig. 7B). TRAP^+^ multinuclear cells were also found within the inflammatory area (Fig. 7B). In FAM-ZOL-injected mice, FAM-positive large cells were found on the palatal bone as well as away from the bone surface (Fig. 7B). Taken together, these cells in the inflammatory area were thought to be ZOL-affected osteoclasts. Immunohistology for CK14 confirmed oral epithelial hyperplasia in the palatal/gingival tissue of ZOL-injected mice (Fig. 7C). Epithelial hyperplasia was primarily observed within the strong inflammatory region. Flow cytometric analysis of dissociated gingival cells demonstrated equal percentages of NK cells, whereas on average the percentages of T cells were somewhat decreased in cells dissociated from ZOL-injected mice when compared to NACL-injected mice, however, such differences were not statistically significant (Fig. 7D). In regards to femur bone marrow (BM) derived cells, although similar percentages of NK cells were obtained, the percentages of T cells were elevated in ZOL-injected mice when compared to NACL-injected mice, however, the differences were not statistically significant (Fig. 7E).

BM derived cells and cells obtained from gingival tissues after injection of ZOL *in vivo* were treated with IL-2 before the levels of IFN-γ secretion were assessed. As shown in Fig. 7F and 7G, injection of ZOL in animals decreased the amounts of IFN-γ secretion by gingiva cells (Fig. 7F) but increased those from BM cells (Fig. 7G). Conversely, NACL-injected animals had higher levels of IFN-γ secretion in gingival associated cells treated with IL-2, but lower induction with IL-2 treated BM cells when compared to ZOL-treated animals (Fig. 7F and 7G). Similarly, IL-2 treated dissociated pancreatic cells and adipose tissue from ZOL-injected animals had lower secretion of IFN-γ when compared to NACL-injected animals (*P* < 0.05) (Fig. 7H). Untreated BM cells, unlike IL-2 treated cells, did not secrete significant levels of IFN-γ (data not shown). Thus, ZOL-induced IFN-γ secretion in different tissue compartments is distinct depending on the location and the type of cells (Fig. 7).

To determine whether IFN-γ is the only cytokine decreased in gingival tissues, we determined the amounts of secreted IL-6, which was also significantly decreased in IL-2 treated gingival tissues of ZOL-injected mice (*P* < 0.05) (Fig. 7I). In addition, *in situ* immunohistochemical analysis of gingival tissues demonstrated strong IL-6 staining at the membrane of osteoclasts in the jawbone of NACL-injected control mice, whereas in ZOL-injected mice it showed a weak and diffuse staining intra-cellularly and no significant staining at the membrane of osteoclasts (Fig. 7J). In contrast to gingiva, secretion of IL-6 was elevated in IL-2-treated pancreatic and BM cells from ZOL-injected mice when compared to NACL-injected mice (Fig. 7K, 7L). In addition, no significant NK cell cytotoxicity could be obtained with either ZOL- or NACL-injected IL-2 treated gingival and adipose dissociated cells, whereas cells dissociated from pancreatic tissues mediated significant cytotoxicity and the levels were similar between ZOL- and NACL-injected mice (Fig. 7M). In contrast, cytotoxicity by BM derived cells was significantly higher in ZOL-injected cells when compared to NACL-injected mice (*P* < 0.05) (Fig. 7N).

In addition, when NK cells were purified from BM cells (BM-NK cells) and used in cytotoxicity assay BM-NK cells treated with IL-2 from ZOL-injected mice had significantly higher cytotoxic activity (*P* < 0.05) (Fig. 7O), and secreted higher levels of IFN-γ (*P* < 0.05) (Fig. 7P) and IL-6 (Fig. 7Q) when compared to NK cells isolated from BM of NACL-injected mice.

Because of significant differences observed between gingival and BM derived cells from ZOL- and NACL-injected mice, we then performed microarray analysis of gingival cells to determine whether 1-suppression of IFN-γ is also observed at the message level, and 2-whether other cytokines, chemokines and growth factors were equally affected (Figs. 7P and S3). Not only the microarray data indicated severe decrease in the IFN-γ and IL-6 levels but many other important genes such as IL-12, IL13, IL15, IL-2 and TNF-α (Fig. 7P) and others (Fig. S3) were decreased, whereas no or slight changes could be seen with CSF2, CXCR3, IL-10 and IL-4 (Fig. 7P). Interestingly, the levels of CTLA-4 gene were enhanced in cells obtained from ZOL-injected gingival cells when compared to NACL-injected mice (Fig. 7R).

## DISCUSSION

Phenotypic and functional characteristics of osteoclasts treated with and without nitrogen-containing BPs: ZOL and ALN, and non-nitrogen containing BP: ETI, were determined in this paper. Initial characterization indicated that during differentiation with RANKL and M-CSF osteoclasts gradually increased the secretion of a number of chemokines and cytokines from day 2 to day 21 of culture, and the profiles of secretion were closer to those of M2 macrophages and monocytes, and different from DCs or M1 macrophages. In comparison to all other subsets, osteoclasts secreted lower amounts of cytokines; however, they secreted substantial amounts of chemokines (Supplemental Table 2). Interestingly, secretion of IL-6 by osteoclasts decreased whereas the IL-10 secretion gradually rose from day 3 of differentiation to day 16. ZOL and ALN, but not ETI, triggered dose dependent secretion of IL-6 whereas they inhibited the secretion of IL-10 by osteoclasts. The dose dependent increase in IL-6 secretion by ZOL was evident when osteoclasts were treated with 10 nM-1 μM. However, at higher concentration of ZOL a dose dependent decrease in the secretion of IL-6 could be observed which related to the ability of ZOL to induce cell death in osteoclasts since after normalization based on the live cells an increase in IL-6 secretion could be observed in doses of 1 μM-50 μM (data not shown). ZOL induced increase in IL-6 was higher when compared to ALN, whereas ETI demonstrated no ability to induce IL-6 secretion. In contrast, IL-10 secretion did not change at lower doses but decreased at higher concentrations of ZOL and ALN but not with ETI. The increase in inflammatory cytokines induced by ZOL and to a lesser extent by ALN correlated with the inability of ZOL and ALN treated osteoclasts to retain their resorptive activity since both the number and size of the pits formed on the resorptive plates were decreased. Interestingly, even though ETI did not induce inflammatory cytokines it was able to decrease the ability of osteoclasts to resorb hydroxyapatite significantly.

To determine differences between monocytes and osteoclasts, a number of key cell surface receptors was analyzed on osteoclasts and compared to monocytes, macrophages and DCs. In comparison to their precursor cell monocytes, osteoclasts expressed much lower levels of MHC class I and II, CD14, CD11b and CD54. Indeed, there was 4–25 fold decrease in the expression of surface receptors on osteoclasts, and the most decrease was seen for MHC class I (8 fold) and II (25 fold) surface expression. The surface expression on osteoclasts was quite different from either macrophages or DCs. Activation of osteoclasts with IFN-γ and TNF-α up-regulated the majority of surface receptors, however, the increase never reached to the levels obtained on the surface of macrophages. These experiments suggested that monocytes in the periphery may be less activating for NK cells since they retain higher expression of MHC class I, whereas once they move to the tissues and down-modulate their surface receptors they may become more activating. Indeed, this may be one reason why NK cells in peripheral blood remain relatively quiescent, even in the presence of competent cytotoxic machinery. Treatment of osteoclasts with ZOL up-regulate surface receptors significantly, and this increase is comparable or even higher when osteoclasts are treated with culture supernatants from highly activated NK cells which are known to increase receptor expression substantially.

In our previous studies we determined that the stage of maturation and differentiation of healthy untransformed stem cells, as well as transformed tumorigenic cancer stem cells, is predictive of their sensitivity to NK cell lysis [5]. Differentiation of stem cells and their resistance to NK cell mediated cytotoxicity correlated with significant increase in the expression of MHC class I, CD54, B7H1 surface expression in a number of healthy and tumor stem cell models, and it was blocked by the addition of the combination of anti-TNF-α and anti-IFN-γ antibodies, which restored NK cell cytotoxicity and blocked the increased expression of above-mentioned surface markers in addition to inhibition of cytokine and chemokine secretion ([39, 40]. Since ZOL increased MHC class I, CD54 and B7H1 on osteoclasts we reasoned that it might behave as a differentiation agent capable of decreasing NK cell mediated cytotoxicity. Indeed, treatment of osteoclasts with ZOL, and much less with ALN, was able to inhibit NK cell cytotoxicity. In addition, NK cells were able to lyse osteoclasts much more than freshly isolated monocytes and this correlated with the decreased expression of MHC class I and CD54 on osteoclasts. Therefore, NK cells can target stem like/non differentiated cells but also they can target differentiated cells, which might have down-modulated or shed those receptors from their surface.

In contrast to the decrease in cytotoxicity, ZOL mediated dose dependent increase in cytokine secretion such as TNF-α (data not shown) and IFN-γ in the co-cultures of NK cells with osteoclasts. As mentioned above, TNF-α and IFN-γ secreted by the NK cells synergistically augmented differentiation of cells resulting in an increase in MHC class I, CD54 and B7H1 and their resistance to NK cell mediated cytotoxicity and decrease in cytokine and chemokine secretion by the NK cells cultured with differentiated cells [40]. It is interesting to note that osteoclasts express lower levels of MHC class I and II and resist upregulation in MHC class I surface expression when either treated with the combination of TNF-α and IFN-γ or with activated NK supernatants known to increase maximally MHC class I and II. This may be one reason why osteoclasts are found to be the best targets for the expansion of fully functional NK cells under the optimized conditions of NK cell stimulation, as shown in this paper. Indeed, such observation is of outmost importance since this strategy may be used to expand NK cells for therapeutic delivery to cancer patients with increased frequencies of cancer stem cells with low/no response to chemo-radiotherapy regimens. Osteoclast-expanded NK cells not only exhibited high cytotoxic activity but also they mediated significant secretion of IFN-γ when compared to DC- and monocyte-stimulated NK cells. Osteoclast-expanded NK cells responded to IL-2 activation and substantially increased IFN-γ secretion per cell basis when compared to NK cells expanded either by DCs or monocytes or secreted by IL-2 treated primary NK cells (data not shown). Interestingly, the addition of ZOL was found to increase MHC class I on osteoclasts, although the mechanisms underlying such increase by ZOL is presently unknown. However, decrease in NK cell cytotoxicity by ZOL is observed after 30 minutes treatment of osteoclasts or tumor cells with ZOL which is quite fast, suggesting that up-regulation of MHC class I expression by ZOL in such a short period of time is less likely event for inhibition of NK cell mediated cytotoxicity. Therefore, ZOL may have a direct effect on inducing resistance of cells to NK cell mediated cytotoxicity. Thus, these results suggest that ZOL-treated osteoclasts may remain viable in the microenvironment for a prolonged period of time and continuously trigger high levels of cytokines and chemokines resulting in the chronicity of inflammation. Indeed, under the conditions in which supernatants from the NK cells induce differentiation in stem cells such as in dental pulp stem cells (DPSCs) or OSCSCs, there is a significant inhibition of both cytotoxicity and cytokine and chemokine secretion [40, 41]. However, in the presence of ZOL, even though NK cell cytotoxicity is inhibited, cytokine secretion continues at a high level, which may be the reason for the adverse effects mediated by ZOL. In addition, ZOL-treated osteoclasts may survive longer and provide continuous NK cell stimulation by the increased production and synergistic functions of NK activating cytokines such as IL-18, IL-15, IL-12 and IFN-α, which we have shown to be secreted by the osteoclasts (Supplemental Table 1 and Fig. 5G). This possibility is under investigation in our laboratory and is the subject of a future report. Both ZOL and ALN but not ETI were able to increase cytokine and chemokine secretion by the NK cells in the co-cultures of NK cells with osteoclast.

To determine whether ZOL could similarly increase IFN-γ secretion locally in tissues and systemically, BM, gingival, pancreatic and adipose tissues was removed and analyzed from mice injected intravenously with ZOL or NACL vehicle solution. Reciprocal increase in the secretion of IFN-γ in BM vs. those of gingival, pancreatic and adipose tissues were observed. Whereas BM cells exhibited higher secretion of IFN-γ when compared to NACL-injected mice, gingival, pancreatic and adipose tissue derived cells decreased IFN-γ in ZOL-injected mice when compared to NACL-injected mice. These observations are of significant value since they may provide potential mechanisms for the pathogenesis seen in osteonecrosis of the jaw (ONJ). It is possible that an increase in activation of BM cells by ZOL result in the eventual loss or inhibition of IFN-γ secreting cells when they reach to the tissues. It has been reported that patients with long-term ZOL treatment exhibit depletion of circulating Vγ9Vδ2 T cells [42, 43]. This clinical observation was postulated to be due to the repeated activations and subsequent depletion of Vγ9Vδ2 T cells through phospho-antigens derived from macrophages responding to ZOL infusion [44]. The present study may suggest that BP-activated osteoclasts in BM may contribute to the over-activation and depletion of a wider array of myeloid as well as lymphoid immune effectors. It is also relevant to note that ETI which does not increase pro-inflammatory cytokine release from osteoclasts does not induce ONJ in patients.

Gingiva-derived immune cells are of activated phenotype when compared to BM-derived immune cells in healthy subjects [45]. Thus, prior activation of BM-derived cells with ZOL when mobilized to the gingival tissues when exposed to the additional activation signals from multitude of antigens including those contributed by more than 450 different species of bacteria in gingiva may undergo activation induced cell death, and become exhausted, resulting in the lower secretion of IFN-γ by the immune cells as seen in our *in vivo* model system. The suppressive effect could be due to lower percentages of T cells but not NK cells in gingiva as observed in mice injected with ZOL, and more importantly due to the suppression of gingival T and NK cell function. Therefore, inability of NK cells to provide key cytokines to activate and expand T cells may be one of the underlying mechanisms for decreased percentages of T cells in the gingival tissues. Conversely, increased function of NK cells in BM as evidenced by higher cytotoxicity and increased induction of IFN-γ could be the reason why an increase in percentages of T cells was seen in BM, although no statistical significance could be obtained for the differences in the percentages of T cells in BM of ZOL- vs. NACL-injected mice. In addition, microarray analysis of gingival tissues from ZOL-injected as compared to NACL-injected mice corroborated findings of lower IFN-γ and IL-6 in addition to several key inflammatory cytokines such as IL-12 and IL-2 and TNF-α which are crucial for the growth and expansion of the immune effectors. Significant inhibition of many inflammatory cytokines, chemokines, co-stimulatory molecules and transcription factors suggests an overall suppressive effect of ZOL injection in gingival tissue immune function. Of note is the no change/slight elevation of IL-10 and IL-4 in microarray analysis. Although these results need to be confirmed at the protein level, the findings may suggest intensified immune-suppressive nature of the oral microenvironment in ZOL-injected mice. It is also of interest to note the elevation of CTLA4 in ZOL-injected oral gingivae, another inhibitory co-stimulatory molecule which is known to dampen the function of activated T cells. The severe decrease observed in chemokines and their receptors is also of a grave concern since this may imply inhibition of immune effector recruitment to the site of pathology, which may deepen immunosuppression. These observations are directly relevant to designing therapeutic strategies to effectively treat ONJ since restoration of immune cell function rather than their inhibition, which has been previously proposed, may be the course of action. Indeed, previous reports of mouse ONJ models applying anti-inflammatory drugs such as dexamethasone have resulted in the exacerbation of the disease [46–48], which would be predicted from our findings.

Oral cavity appears to have the most severe immunosuppression since pancreatic tissues even though expressed lower IFN-γ secretion, they contained higher secretion of IL-6 whereas both IFN-γ and IL-6 was severely inhibited in the oral tissues. Indeed, *in situ* analysis of the osteoclasts in the oral gingival tissues after ZOL injection also corroborated our findings in that these cells exhibit lower membrane expression of IL-6 as compared to NACL-injected oral tissues, and the pattern of staining for IL-6 in ZOL injected oral tissues are more diffuse throughout the osteoclasts. Moreover, osteoclasts were clearly a reservoir of ZOL since they up took fluorescent-labeled ZOL significantly, in addition to being in the connective tissue rather than in the interphase of bone and connective tissue, which healthy osteoclasts are usually found. Another characteristic feature of ZOL-injected mice was the finding of pseudomembranous epithelial hyperplasia (PEH), which is also seen in patients with ONJ. Such feature may distantly resemble that observed in dysplastic and neoplastic tumors; albeit with completely distinct mechanisms since in both cases the local immune effector function is unable to limit proliferation of epithelial cells. Indeed, we have established that NK cells are important effectors, which shape the fraction of stem cells in healthy as well as transformed tissues, and their functional inactivation may be the reason for the progression and establishment of PEH. These possibilities are under investigation in our laboratory. The phenotype and nature of immune cells, including NK cells, one of the main contributors of IFN-γ secretion in bone marrow and gingival tissues are currently being investigated in our laboratory.

The significance and function of monocyte/macrophages and DCs in driving an effective immune response has been known for decades; however, their close relative, osteoclasts were primarily known for their function during bone turn-over and remodeling. Our studies impart greater significance to this subset, and place them in the ranks of monocyte/macrophages and DCs in regulating the function of innate immunity and particularly NK cells.

## MATERIALS AND METHODS

### Cell Lines, reagents, and antibodies

Alpha-MEM medium (Life Technologies, CA) supplemented with 10% Fetal Bovine Serum (FBS) and penicillin-streptomycin (Gemini Bio-Products, CA) was used to culture human osteoclasts. RPMI 1640 supplemented with 10% FBS was used for the cultures of human and murine immune cells. Oral Squamous Carcinoma Stem Cells (OSCSCs) were isolated from oral cancer patient (tongue tumor) at UCLA and were cultured in RPMI 1640 supplemented with 10% FBS (Gemini Bio-Products, CA), 1.4% antibiotic antimycotic, 1% sodium pyruvate, 1.4% non-essential amino acids, 1% L-glutamine, 0.2% gentamicin (Gemini Bio-Products, CA) and 0.15% sodium bicarbonate (Fisher Scientific, PA). Human M-CSF (Biolegend, CA) and soluble RANKL (PeproTech, NJ) were dissolved in alpha-MEM and stored at −80°C. ZOL, ALN and ETI were purchased from UCLA Ronald Reagan Pharmacy. Fluorescent-conjugated ZOL analogs were synthesized via a linker strategy [32]. PE- and FITC-conjugated isotype control, CD3, CD11b, CD14, CD15, CD33, CD44, CD45, CD54, CD124, B7H1, MHC-I and MHC-II were purchased from Biolegend (San Diego, CA). Recombinant IFN-γ and TNF-α were obtained from Biolegend (San Diego, CA). Recombinant IL-2 was obtained from NIH-BRB. Purified antibodies to CD14 and CD16 were purchased from Biolegend (San Diego, CA). Propidium iodide was purchased from Sigma Aldrich (Buffalo, NY). AJ2, prepared in the laboratory, is a combination of 8 sonicated gram positive bacteria with superior ability to induce optimal secretion of both pro-inflammatory and anti-inflammatory cytokines in NK cells (manuscript submitted).

### Purification of monocytes and generation of dendritic cells and osteoclasts from mice and humans

Written informed consents approved by UCLA Institutional Review Board (IRB) were obtained from healthy blood donors and all the procedures were approved by the UCLA-IRB. Peripheral blood mononuclear cells (PBMCs) from healthy donors were isolated as described before [33]. Briefly, PBMCs were obtained after Ficoll-hypaque centrifugation. PBMCs were added to the tissue culture plates, after which the adherent sub-populations of PBMCs were detached and the monocytes were negatively purified using isolation kits obtained from Stem Cell Technologies (Vancouver, Canada). Murine monocytes were also negatively isolated from bone marrow cells. Greater than 95% purity was achieved for each subset based on flow cytometric analysis of CD14. Purified monocytes were cultured in alpha-MEM medium containing M-CSF (25 ng/mL) and RANKL (25 ng/mL) for 21 days, or otherwise specified. Medium was refreshed every 3 days with fresh alpha-MEM containing M-CSF and RANKL. Purified monocytes were cultured in RPMI medium containing GM-CSF (150 ng/mL) and IL-4 (50 ng/mL) for 7 days to generate dendritic cells.

### Purification of human NK cells and murine NK cells, T cells and γδ T cells

PBMCs from healthy donors were isolated as described before [33]. Briefly, peripheral blood lymphocytes were obtained after Ficoll-hypaque centrifugation and purified NK cells were negatively selected by using an NK cell isolation kit (Stem Cell Technologies, Vancouver, Canada). The purity of NK cell population was found to be greater than 90% based on flow cytometric analysis of PE conjugated anti-CD16 antibody stained cells. The levels of contaminating CD3^+^ T cells remained low, at 2.4% ± 1%, similar to that obtained by the non-specific staining using isotype control antibody throughout the experimental procedures. Similarly, murine NK and T cells were negatively purified using isolation kits from stem cell technologies (Stem Cell Technologies, Vancouver, Canada) whereas murine γδ T cells were negatively selected using isolation kit from Miltenyi biotech (Miltenyi, biotech; CA) from mouse splenocytes. The purity of murine NK and T cells was greater than 90% based on staining with DX5 and CD3, whereas the purity of murine γδ T cells was greater than 80% based on staining with GL3 antibody.

### TRAP staining

The staining was carried out as recommended by the manufacturer (Sigma-Aldrich). Briefly, osteoclasts were detached from tissue culture plates and seeded in 96-well plate at 3 × 10^4^ cells/well for 18–24 hours. Afterwards the cells were rinsed twice with 1X PBS and fixed with the fixative solution containing citrate solution, acetone and 37% formaldehyde. Cells were then washed twice with 1XPBS before Fast Garnet GBC and sodium nitrite at (1:1 ratio) were added and incubated for 1 hour at 37° C. Plates were rinsed and hematoxylin were then added to each well for 2 mins. Plates were air dried and images were taken by the Leica DMI 6000B inverted microscope.

### Pit resorption assay

Osteoclasts differentiated from monocytes for 21 days were rinsed, detached from tissue culture plates and seeded at 1 × 10^4^ cells/well in 24 well plate pre-coated with synthetic carbonate apatite (Cosmo Bio Co, Japan) for 7 days. After the incubation period, culture medium was removed and cells were rinsed with freshly prepared 5% sodium hypochlorite for 5 minutes. The cells were then washed with water and photographed using Leica DMI 6000B inverted microscope.

### ELISAs

ELISAs were performed as described previously [33]. To determine the cytokine concentrations to obtain the cytokine concentration, a standard curve was generated by either two or three fold dilution of recombinant cytokines.

### Multiplex cytokine array kit

Fluorokine MAP cytokine multiplex kits were purchased from Life Technologies (Carlsbad, CA) and the procedures were conducted as suggested by the manufacturer. To determine the cytokine concentrations a standard curve was generated by threefold dilution of recombinant cytokines provided by the manufacturer. Analysis was performed using MAGPIX (Life Technologies, CA).

### Surface staining

Staining was performed by labeling the cells with antibodies as described previously [33–35]. Flow Cytometry analysis was performed using Beckman Coulter Epics XL cytometer (Brea, CA) and results were analyzed was FlowJo vX software (Ashland, OR).

### ^51^Cr release cytotoxicity assay

The ^51^Cr release assay was performed as described previously [36]. Briefly, different numbers of purified NK cells were incubated with ^51^Cr–labeled target cells. After a 4 hour incubation period the supernatants were harvested from each sample and counted for released radioactivity using the gamma counter. The percentage specific cytotoxicity was calculated as follows:

$$\%\text{ Cytotoxicity}=\frac{\text{Experimental cpm}-\text{spontaneous cpm}}{\text{Total cpm}-\text{spontaneous cpm}}$$

LU 30/10^6^ was calculated by using the inverse of the number of effector cells needed to lyse 30% of target cells X100.

### ZOL injection and tooth extraction in mice

The UCLA Animal Research Committee reviewed and approved all experimental protocols involving animals (ARC# 1997–136). Seven weeks old female Balb/c mice (Jackson Laboratory, Bar Harbor, ME) received a bolus injection of 184 μM ZOL (500 μg/Kg) or 184 μM 5-Carboxyfluorescein (FAM)-conjugated ZOL in 200 μl 0.9% NACL solution from tail vein. A separate group was injected with 0.9% NACL vehicle solution. One week later, maxillary first molar was extracted. Mice were anesthetized via isoflurane inhalation and placed on a custom-made surgical table. After the circumferential periodontal ligament of the attached gingiva was dissected, the maxillary left first molar was luxated by a dental explorer and gently removed using surgical forceps. Immediately prior to tooth extraction, 5.0 mg/kg carprofen was subcutaneously injected, and this injection was repeated every 24 hours for 48 hours. After tooth extraction, mice were fed gel diet (DietGel 76A, ClearH_2_O, Portland, ME).

### Histological characterization of mouse maxillary tissue

One week and two weeks after tooth extraction, mice were euthanized by 100% CO_2_ inhalation and the maxillary tissue containing the wound healing site was harvested and digitally photographed. Then the maxillary tissue was fixed in 10% buffered formalin overnight and decalcified in 10% EDTA for 7 to 10 days. Histological sections of paraffin-embedded histological specimens were generated in a frontal plane through the tooth extraction site and stained by hematoxylin and eosin (H & E) or for Tartrate-resistant acid phosphatase (TRAP Kit, Sigma-Aldrich, St. Louis, MO). Adjacent histological sections were subjected to antigen retrieval via microwave irradiation and incubated with an anti-cytokeratin 14 or an anti-IL-6 antibody (Santa Cruz Biotechnology, Dallas, TX, USA) followed by incubation with diaminobenzidine substrate. The sections were counterstained with hematoxylin.

The maxillary bone of FAM-ZOL-injected mice was examined by the standardized fluorescent biophotonics imager using an excitation wavelength of 460 nm and the 515-nm filter (LAS3000, FUJIFILM Corp, Tokyo, Japan). The maxillary specimens with palatal/gingival tissue of FAM-ZOL-injected mice were fixed in 70% ethyl alcohol overnight and embedded in plastic resin (methyl methacrylate, Polysciences, Warrington, PA; dibutyl phthalate and benzoyl peroxide, Sigma Aldrich, St. Louis, MO) following the manufacturer's protocol. Frontal sections were made through tooth extraction site and FAM-ZOL was detected by a fluorescent microscope.

### Cell dissociation from femur, spleen, pancreas, peri-pancreatic fat and gingiva of ZOL-injected mice

Female C57Bl/6J B6 mice received IV injection of 500 μg/Kg ZOL or 0.9% NACL vehicle solution followed by maxillary first molar extraction after 7 days as described above. All mice were euthanized two weeks after tooth extraction. After euthanasia, gingival tissues, pancreas and peri-pancreatic fat were obtained from ZOL and NACL injected animals and digested using collagenase II (1 mg/ml) (gingival tissue and peri-pancreatic fat) and collagenase IV (1 mg/ml) (pancreas) (Invitrogen, CA) and DNAse (10 units/ml) (Sigma-Aldrich, CA) for 20 minutes at 37°C. Femurs from ZOL and NACL injected animals were harvested and bone marrow cells were extracted and cultured in the presence and absence of IL-2 (10, 000 units/ml) treatment using RPMI 1640 media (Life technologies, CA). Single cells released from the gingival tissues, peri-pancreatic fat and pancreas were suspended in media and cultured in the presence of IL-2 (10,000 units/ml).

### Cytokine/chemokine gene expression in the mouse maxillary palatal/gingival tissues

One week after tooth extraction, the maxillary gingival tissue was harvested from mice pre-injected with ZOL (*n* = 2) or 0.9% NACL vehicle solution (*n* = 2). The tissue was snap frozen in liquid nitrogen and total RNA was prepared using a commercially available kit (RNeasy Fibrous Tissue Mini Kit, Qiagen, Valencia, CA). A reference RNA sample was prepared from the gingival tissue of a naïve mouse. The steady state mRNA levels of cytokines and chemokines in the palatal/gingival tissue were evaluated by a commercially available PCR array (RT^2^ Profiler^TM^ PCR Array Mouse Th1&Th2 Responses, Qiagen, Valencia, CA) following the manufacturer's protocol. The relative gene expression value was calculated by ΔΔCt using the internal housekeeping gene and the reference gene expression.

### Statistical analysis

An unpaired, two-tailed student *t*-test was performed for the statistical analysis. One way ANOVA with a Bonferroni post-test was used to compare the different groups.

## SUPPLEMENTARY FIGURES AND TABLES


