# Erratum: Bisphosphonate-induced differential modulation of immune cell function in gingiva and bone marrow *in vivo*: Role in osteoclast-mediated NK cell activation

**DOI:** 10.18632/oncotarget.6431

**Published:** 2015-11-29

**Authors:** Han-Ching Tseng, Keiichi Kanayama, Kawaljit Kaur, So-Hyun Park, Sil Park, Anna Kozlowska, Shuting Sun, Charles E. McKenna, Ichiro Nishimura, Anahid Jewett

Oncotarget. 2015; 6:20002-20025

**PMID**: 26343372

**Present**:

Conflict of Interests is missing in the original paper.

**Correction**:

The authors declare no Conflict of Interests.

