# Supplementary material for: Bisphosphonate-induced differential modulation of immune cell function in gingiva and bone marrow in vivo: Role in osteoclast-mediated NK cell activation
Source: Oncotarget. 2015 Jul 28;6(24):20002–25. doi: 10.18632/oncotarget.4755 (PMC4652983; doi:10.18632/oncotarget.4755)
Supplement: Supplementary file 1 [file oncotarget-06-20002-s001.pdf]

SUPPLEMENTARY FIGURES AND TABLES

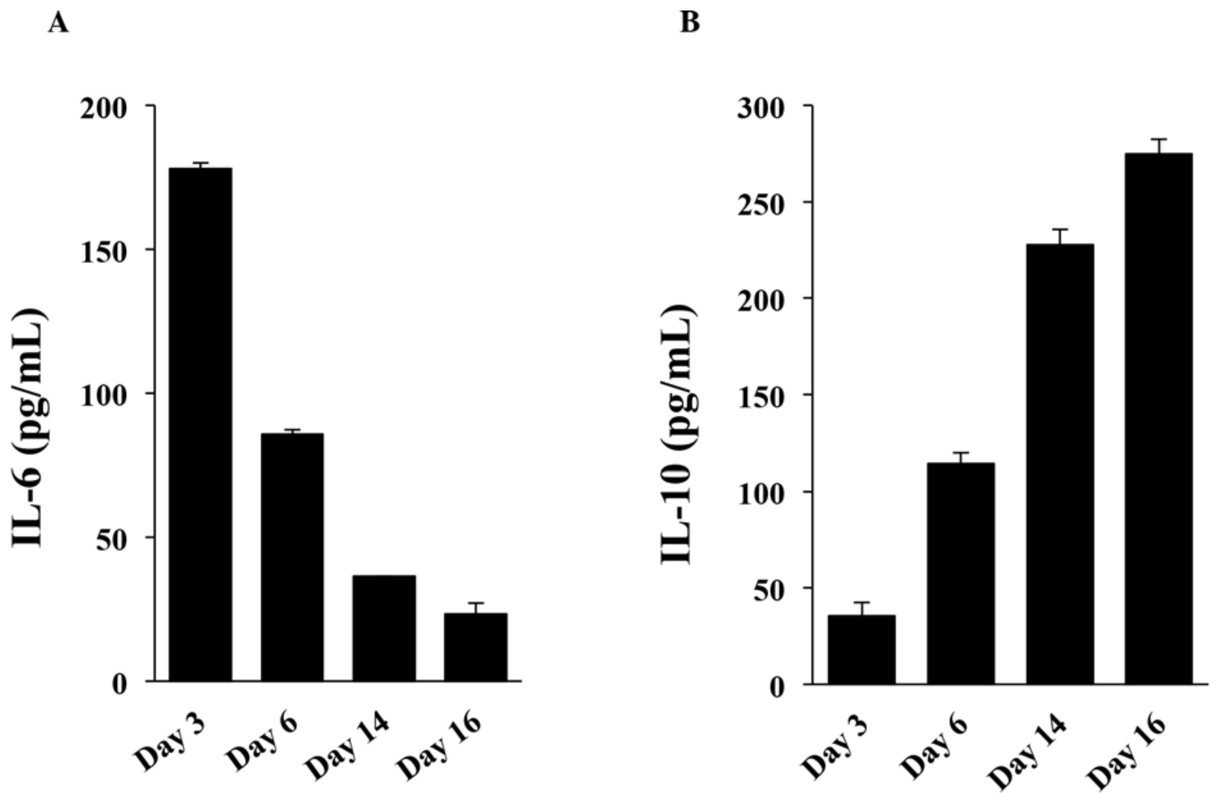

M-CSF	+	+	+	+
RANKL		+	+	+

M-CSF	+	+	+	+
RANKL		+	+	+

Supplementary Figure S1: Decrease in IL-6 and increase in IL-10 production during osteoclast differentiation from monocytes. A. Osteoclasts were generated from monocytes as described in the Materials and Methods section for 16 days. Culture medium was collected at indicated time points and the levels of IL-6. and **B.** IL-10 secreted by osteoclasts were measured using specific ELISAs.

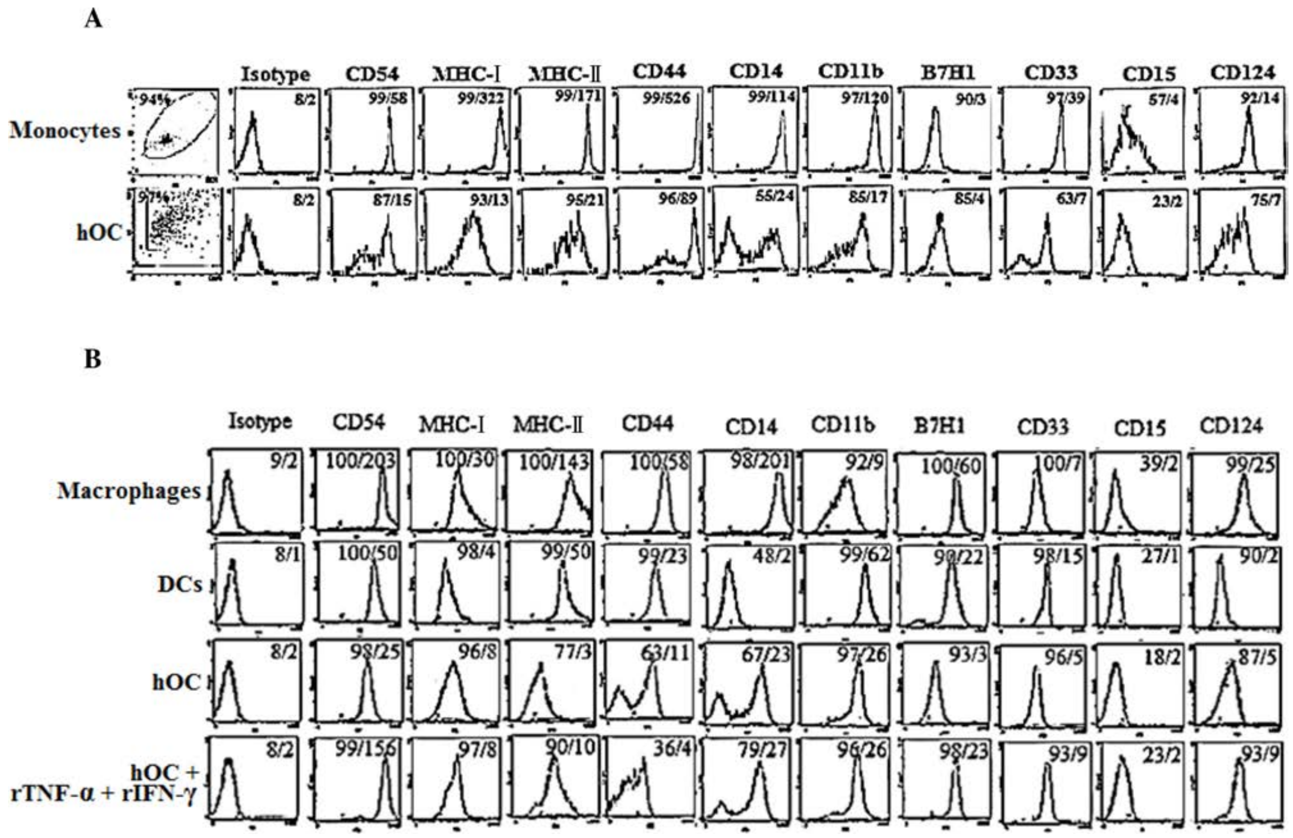

Supplementary Figure S2: Surface expression profiles of monocytes, osteoclasts, macrophages and DCs. A. Osteoclasts were generated from purified autologous monocytes as described in the Materials and Methods section. Surface expression of CD54, MHC-I, MHC-II, CD44, CD14, CD11b, B7H1, CD33, CD15 and CD124 on monocytes and osteoclasts were assessed with flow cytometric analysis after staining with the respective PE-conjugated antibodies. Isotype control antibodies were used as control. The numbers on the right hand corner are the percentages and the mean channel fluoresence intensities for each histogram. **B.** Monocytes, DCs and osteoclasts were generated as described in Supplemental table 2. On the last day of differentiation, osteoclasts were left untreated or treated with rh-IFN-γ (10 ng/ml) and rh-TNF-α (20 ng/ml) for 24 hours. Afterwards, the surface expression of CD54, MHC-I, MHC-II, CD44, CD14, CD11b, B7H1, CD33, CD15 and CD124 were assessed with flow cytometric analysis after staining with the respective PE-conjugated antibodies. Isotype control antibodies were used as control. The numbers on the right hand corner are the percentages and the mean channel fluoresence intensities for each histogram.

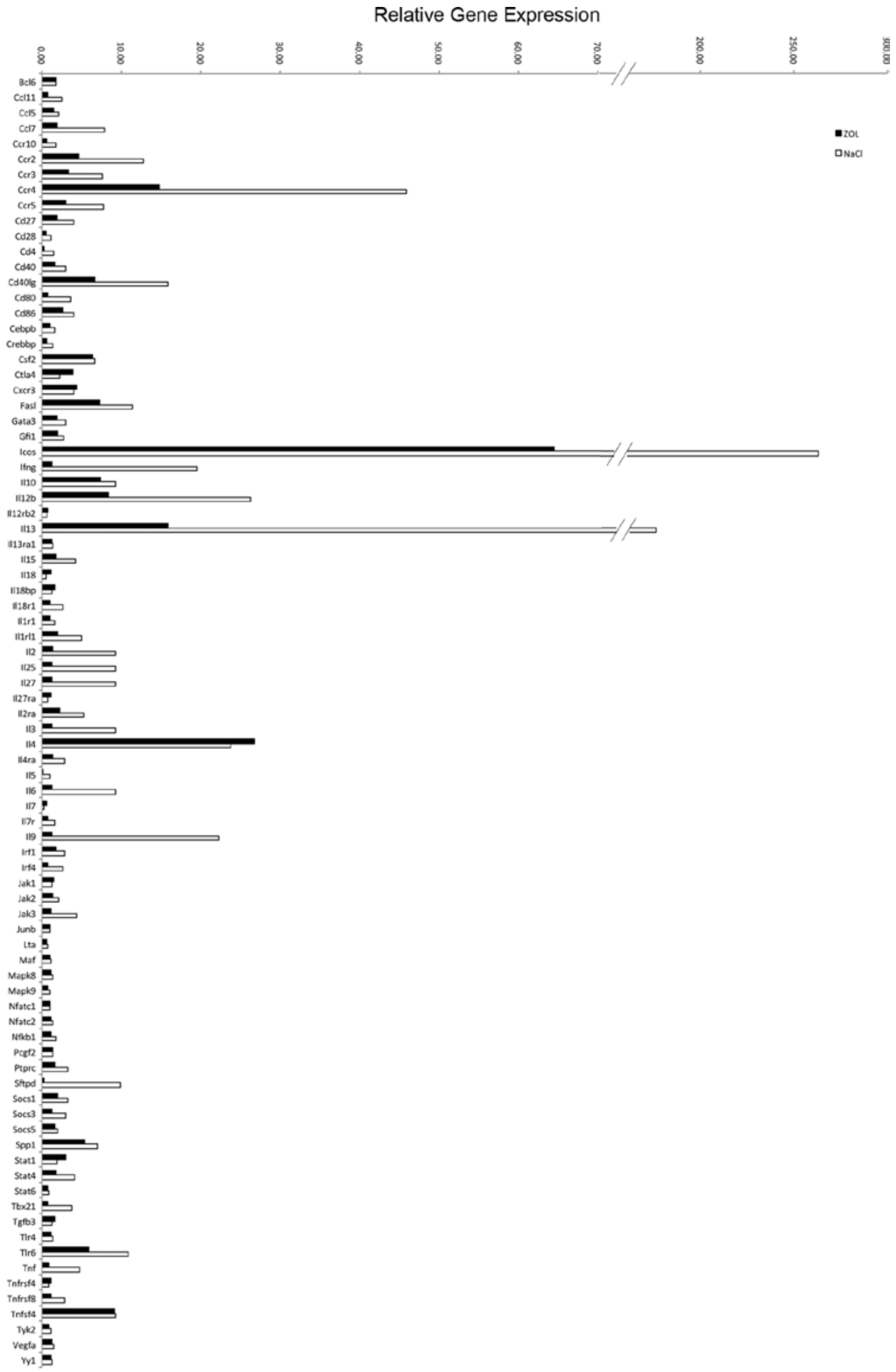

Supplementary Figure S3: Microarray analysis of pro-inflammatory cytokines, chemokines, growth factors and transcription factors of gingival tissues after intravenous ZOL and NaCl injection. A commercially available PCR microarray (RT^2 Profiler™ PCR Array Mouse Th1&Th2 Responses, Qiagen, Valencia, CA) was used to characterize the steady state mRNA levels of cytokines and chemokines expressed in the tooth extraction wound of palatal/gingival tissues in mice pre-treated with ZOL ($n = 2$) or 0.9% NaCl vehicle solution ($n = 2$). Total RNA sample of palatal/gingival tissue from a naïve mouse served as a reference control. The data of each entry was averaged in the group.

Supplementary Table S1. Production of cytokines, chemokines and growth factors by osteoclasts

Concentration (pg/mL)					
	Day 2	Day 6	Day 10	Day 14	Day 21
IL-1RA	680	673	3168	4897	4342
IL-2R	31	31	65	156	212
IL-12	20	8	17	24	32
IL-15	59	72	163	79	52
IFN-α	90	108	126	106	69
IFN-γ	0	0	0	0	0
IL-6	108	14	58	34	16

Concentration (pg/mL)					
	Day 2	Day 6	Day 10	Day 14	Day 21
MIP-1α	40	43	640	1932	5071
MIP-1β	116	109	1914	3908	5168
RANTES	271	55	63	238	432
MCP-1	>6100	>6100	>6100	>6100	3628
IL-8	20002	24988	22072	ND	22267
MIG	0	2	28	12	14
IP-10	16	24	56	19	13
Eotaxin	0	0	0	0	0

Human monocytes purified from healthy donor's PBMCs were differentiated into osteoclasts as described in the Materials and Methods section. Culture medium was collected on days 2, 6, 10, 14 and 21 of culture and the levels of cytokine and chemokine production were measured using multiplex cytokine array kit.

Supplementary Table S2. Method of generation and production of cytokines, chemokines and growth factors by monocytes, dendritic cells, M1 macrophages, M2 macrophages and osteoclasts

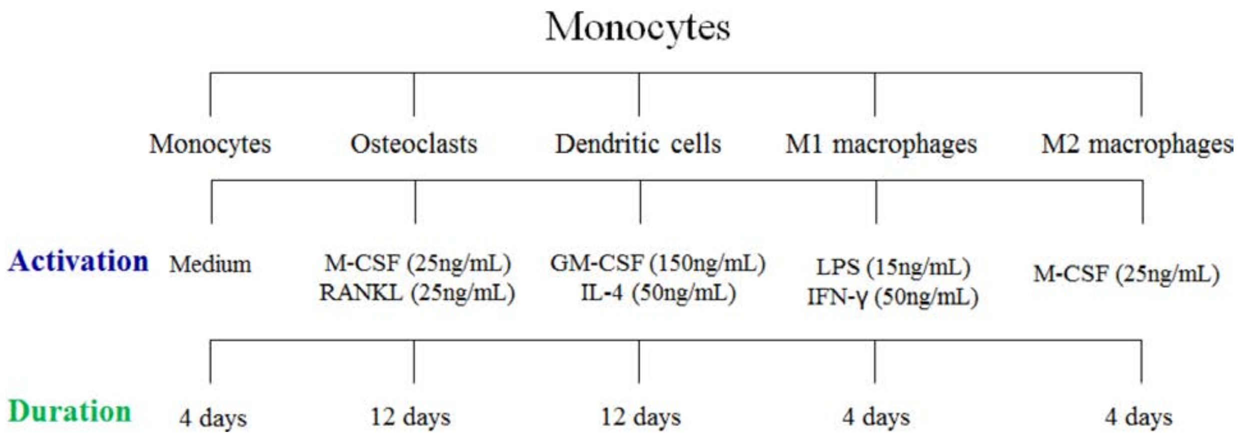

	IL-1b	IL-1Ra	IL-2	IL-4	IL-5	IL-6	IL-7	IL-9	IL-10	IL-12p70	IL-13	IL-15	IL-17	IFN-y	TNF-α
**Monocytes**	8	159	13	3	0	293	3	12	21	24	0	17	42	0	26
**DCs**	10	9397	22	NA	4	687	4	11	87	6	5	18	72	0	47
**M1 macrophages**	>1952	4434	35	6	0	32200	14	15	1081	73	12	0	70	NA	710
**M2 macrophages**	15	434	20	4	4	395	8	18	33	33	4	20	76	0	44
**Osteoclasts**	0	91	11	2	0	46	0	10	14	10	0	21	32	0	24

	MCP-1	MIP-1 α	MIP-1 β	RANTES	Eotaxin	IL-8	IP-10
**Monocytes**	1152	18	205	>1891	0	7210	619
**DCs**	111	21	147	>1891	0	3110	175
**M1 macrophages**	94	207	2100	673	34	22262	481
**M2 macrophages**	3002	20	298	>1891	0	27886	1878
**Osteoclasts**	2133	17	187	1560	0	3874	1285

	Basic FGF	VEGF	PDGF-BB	G-CSF	GM-CSF
**Monocytes**	0	151	281	66	85
**DCs**	0	19	325	86	NA
**M1 macrophages**	0	996	338	9349	53
**M2 macrophages**	0	251	425	81	62
**Osteoclasts**	0	76	117	31	90

Highly purified human monocytes were left untreated or treated with different stimuli as shown above. Supernatants were harvested from each sample on the indicated days and the levels of cytokines and chemokines were measured using multiplex cytokine array kit. Dendritic cells (DCs). Not available (NA).
